# Exploiting the Legacy of the Arbovirus Hunters

**DOI:** 10.3390/v11050471

**Published:** 2019-05-23

**Authors:** Nikos Vasilakis, Robert B. Tesh, Vsevolod L. Popov, Steve G. Widen, Thomas G. Wood, Naomi L. Forrester, Jean Paul Gonzalez, Jean Francois Saluzzo, Sergey Alkhovsky, Sai Kit Lam, John S. Mackenzie, Peter J. Walker

**Affiliations:** 1 Department of Pathology, University of Texas Medical Branch, 301 University Blvd, Galveston, TX 77555, USA; rtesh@utmb.edu (R.B.T.); vpopov@utmb.edu (V.L.P.); naforres@utmb.edu (N.L.F.); 2 Center for Biodefense and Emerging Infectious Diseases, University of Texas Medical Branch, 301 University Blvd, Galveston, TX 77555, USA; 3 Institute for Human Infection and Immunity, University of Texas Medical Branch, 301 University Blvd, Galveston, TX 77555, USA; 4 Center for Tropical Diseases, University of Texas Medical Branch, 301 University Blvd, Galveston, TX 77555, USA; 5 Department of Biochemistry and Molecular Biology, University of Texas Medical Branch, 301 University Blvd, Galveston TX 77555, USA; sgwiden@utmb.edu (S.G.W.); tgwood@utmb.edu (T.G.W.); 6 Center of Excellence for Emerging & Zoonotic Animal Disease, Kansas State University, Manhattan, KS 66502, USA; jpgonzalez@vet.k-state.edu; 7 Oncovita, Institut Pasteur, 25-28 Rue du Dr Roux, 75015 Paris, France; jfsaluzzo@gmail.com; 8 Ivanovsky Institute of Virology, N.F. Gamaleya National Research Center for Epidemiology and Microbiology, Ministry of Healthcare of the Russian Federation, 123098, 18 Gamaleya str., Moscow, Russia; salkh@yandex.ru; 9 Department of Medical Microbiology, University of Malaya, Kuala Lumpur 50603, Malaysia; lamsk@nipahvirus.org; 10 Faculty of Medical Sciences, Curtin University, Perth, Western Australia 6102, Australia; J.Mackenzie@curtin.edu.au; 11 School of Biological Sciences, The University of Queensland, St Lucia, QLD 4072, Australia

**Keywords:** arbovirus history, arbovirus discovery, next generation sequencing, electron microscopy, metagenomics, taxonomy

## Abstract

In recent years, it has become evident that a generational gap has developed in the community of arbovirus research. This apparent gap is due to the dis-investment of training for the next generation of arbovirologists, which threatens to derail the rich history of virus discovery, field epidemiology, and understanding of the richness of diversity that surrounds us. On the other hand, new technologies have resulted in an explosion of virus discovery that is constantly redefining the virosphere and the evolutionary relationships between viruses. This paradox presents new challenges that may have immediate and disastrous consequences for public health when yet to be discovered arboviruses emerge. In this review we endeavor to bridge this gap by providing a historical context for the work being conducted today and provide continuity between the generations. To this end, we will provide a narrative of the thrill of scientific discovery and excitement and the challenges lying ahead.

## 1. Introduction

Almost 120 years have passed since Walter Reed, James Carroll, Aristides Agramonte, and Jesse Lazear established that yellow fever is caused by a filterable infectious agent which is transmitted by the bite of a mosquito, then known as *Stegomyia fasciata* (*Aedes aegypti*). Lazear, who like his colleagues, had been stationed by the US Army in Cuba to study the disease, died of yellow fever in September 1900 after being exposed experimentally to mosquitos that had fed on sick patients. At about the same time in South Africa, James Spreull and Sir Arnold Theiler demonstrated that bluetongue disease of sheep is caused by an “ultravisible” agent that could be transmitted by the injection of an infected serum. Epidemiological evidence suggested that the agent was vector-borne, and it was subsequently shown by R.M. du Toit that the disease occurred in sheep inoculated experimentally with suspensions of wild-caught biting midges (*Culicoides imicola*). These and other seminal discoveries precipitated a century of research into vector-borne and zoonotic viral diseases, resulting in the discovery and isolation of many hundreds of novel viruses from insects or vertebrate hosts. Some were identified as important human or veterinary pathogens. Many other viruses were archived in reference collections, with only basic characterization of their biological or molecular properties. In recent years, the advent of next generation sequencing (NGS) has transformed this situation. Complete genome sequences are now available for many of the archived isolates, allowing more accurate taxonomic assignments, analysis of their phylogenetic and evolutionary relationships with other viruses, and evaluation of the potential risks they may present to humans and wild or domestic animal populations. NGS has also opened the door to viral metagenomics, which has greatly increased the pace of new virus discovery from a wide range of hosts, usually with complete or near-complete viral coding sequences, but no virus isolate and minimal biological data. This has presented both opportunities and challenges for virologists and epidemiologists, as well as viral taxonomists, evolutionary biologists, and bioinfomaticians. Sadly, this technological revolution has been accompanied by a period of progressive disinvestment in training in classical virology. In this review, we recall the rich history of the discovery of arboviruses and other zoonotic viruses in various settings around the world and the many outstanding scientists who have contributed to the endeavour. We also consider the impacts of NGS and metagenomic analysis, and the implications of these new technologies for the future of this important field of research.

## 2. History of Arbovirus Discovery

### 2.1. Rockefeller Foundation and Yale Arbovirus Research Unit (YARU)

The Rockefeller Foundation (RF) was organized in 1913 for “the well-being of mankind throughout the world” [1]. At the time, yellow fever was still epidemic in many tropical and subtropical regions of Africa and the Americas, so in 1916, the RF established the Yellow Fever Commission, with the lofty goal of eradicating yellow fever from the world. During the next 25 years, the RF supported an international group of scientists working on yellow fever in New York City in the United States (U.S.); Rio de Janeiro and Salvador in Brazil; Bogota, Colombia; Yabba, Nigeria; and Entebbe, Uganda [1,2]. Much of the work and accomplishments of RF-funded personnel during this period was described in Strode’s classic book, entitled “Yellow Fever” [3]. The major accomplishments included:

1. Confirmation of earlier work by the Reed Commission in Cuba, demonstrating that yellow fever was caused by a filterable agent, yellow fever virus (YFV), that was transmitted by the bite of infected mosquitoes, *Aedes aegypti*;
2. Discovery that the rhesus monkey and the white mouse are susceptible to infection with YFV, providing models for subsequent studies on the pathogenesis, transmission, epidemiology, and control of the disease;
3. Demonstration that convalescent sera of humans and animals infected with YFV neutralize the virus. This discovery led to the development of the mouse neutralization test, which allowed investigators to map the geographic distribution of the virus;
4. Discovery of the forest or sylvan cycle of YFV and the importance of mosquitoes other than *Ae. aegypti* in the transmission and maintenance of the virus;
5. Development of the 17D vaccine strain of YFV and its first human trials. Max Theiler, son of South African bluetongue researcher Sir Arnold Theiler, received a Nobel Prize in 1951 for this work.

As a by-product of the overseas yellow fever investigations, RF-funded researchers also isolated a number of other previously unknown arboviruses, including West Nile, Zika, Semliki Forest, Bunyamwera, Bwamba, Uganda S, Ilheus, and Anopheles A and B viruses [2].

The threat and onset of World War II changed the priorities of the RF, and most of its overseas YF research activities ceased during this period. Many of the former American RF staff became involved in studies of diseases of military importance, such as typhus, malaria, sandfly fever, and dengue, some as civilians and others as members of the U.S. Armed Forces.

In 1950, after the end of the war, the RF decided to develop a major new program to study arthropod-borne viruses and to discover “what might be out there”, as well as their disease associations, life cycles, and vectors. This led to the development of the Rockefeller Foundation Virus Program, a world-wide virus discovery program that was funded from 1951 to 1970 [1]. Over the next several years, field laboratories staffed by both RF and local scientists were established with foreign governments in Pune, India; Port of Spain, Trinidad; Belem, Brazil; Johannesburg, South Africa; Ibadan, Nigeria; Cali, Colombia; and Cairo, Egypt [2]. In Egypt, the Cairo laboratory was associated with the U.S. Naval Medical Research Unit No. 3. Scientists in these field laboratories were involved in the detection and investigation of human diseases in their respective geographic regions, surveying human and animal populations for serologic evidence of past viral infection, and searching for viruses in a wide variety of arthropods, mammals, birds, reptiles, and amphibians [2]. All virus isolations were done on site, using the classic technique of intracranial inoculation of newborn white mice, but later, as vertebrate cell cultures became available, inoculation of cell cultures was also used. In addition, sentinel animals, such as non-human primates, mice, and hamsters, were also used in the field to detect virus activity. Viruses isolated in the overseas laboratories were initially characterized locally, lyophilized for preservation and storage, and then aliquots of each new virus were shipped back to the RF central virus laboratory in New York for further characterization and study. It was during this period that Jordi Casals, Delphine Clarke, Loring Whitman, and others developed the sucrose-acetone method for preparation of viral antigens and began to adapt the hemagglutination inhibition (HI) and complement fixation (CF) tests to identify and group arboviruses [4,5,6].

The RF Virus Program was extremely productive and many novel arboviruses, as well as non-arthropod-borne viruses (i.e., hantaviruses and arenaviruses), were discovered by RF-funded investigators during this period. The productivity of this search strategy in detecting novel viruses affecting humans was recently reviewed by Rosenberg et al. [7]. The virus discovery rate was highest in the period 1950–1969, which coincided with the RF Virus Program. A similar approach was also practiced by the Institut Pasteur and other international groups involved in virus discovery during this period, as described in this article and in other publications [8,9,10,11,12,13], resulting in the detection of a high proportion of pathogenic arboviruses. The second advantage of the strategy was that it yielded actual virus isolates, whose pathogenesis could be studied experimentally in vivo and in vitro in vertebrate and arthropod models. In contrast, current search methods for new viruses, which generally use metagenomics and other sophisticated genetic techniques to detect novel viral agents, do not usually yield live viruses, only their nucleotide sequences. A complete or partial genetic sequence alone rarely provides insight into the epidemiology and ecology, host range, pathogenesis and disease potential, or transmission modes of the new viruses.

In 1960, the RF made a decision to phase-out its Virus Program and to devote more of its efforts and resources to other projects, such as population control and increasing food production (“the green revolution”). Over the next 10 years, the RF-funded investigators working in overseas laboratories were withdrawn and the respective laboratories were turned over to local institutions or governments. In 1964, the RF made arrangements with Yale University to transfer its Arbovirus Group to New Haven. Accordingly, the Virus Laboratory in New York City was closed, and most of the remaining investigators (Casals, Clarke, Downs, Theiler, Buckley, Shope, Aitken, etc.), their equipment, virus stocks, and reagents were moved to New Haven, with a small endowment to temporarily support them. The newly established group was designated the Yale Arbovirus Research Unit (YARU) and was housed in the newly constructed Laboratory of Epidemiology and Public Health building on the grounds of the Yale University School of Medicine. Upon arrival, the former RF staff members in turn became members of the Yale faculty [1,2]. Wilbur Downs was designated as the first Director of YARU, and in 1974 when Downs retired, Robert E. Shope became the Director.

The YARU was subsequently designated by WHO as an International Reference Center for Arboviruses, and for a number of years, it received virus samples from investigators worldwide for identification and confirmation [14]. Under Shope’s leadership, the World Reference Center for Arboviruses (WRCA) was established. Initially, it consisted of the original viruses (known and unknown) that came along with the staff from the former RF Virus Laboratory in New York, but over the years, as new viruses were submitted for study or were isolated by YARU investigators, they were also added to the WRCA collection [14]. As the former RF personnel retired, new researchers were recruited to YARU as full Yale University faculty [1]. Because of its prestige and university affiliation, many visiting scientists, graduate students, and other trainees from the U.S. and a variety of other countries came to YARU during this period to learn arboviral techniques, to do original research, and to identify samples collected by them during outbreaks or field studies. These samples were also added to the WRCA collection. Many of these visitors, as well as some of the younger YARU faculty, eventually moved on to assume prominent positions in academia, government service, or the corporate world. Shope [14] and Downs [1] have described some of the significant activities, accomplishments, and international collaborations during the early years of YARU.

Without continued support from the RF or from Yale University, the YARU staff, like other university scientists in the U.S., had to compete for grants and contracts to support their research. This eventually changed the nature of YARU’s research activities from international outbreak investigation, diagnostic support, and training to investigator-initiated, hypothesis-driven grant research or contract activities. Meanwhile, Shope and other YARU staff members continued to serve as consultants to U.S. Government agencies [National Institutes of Health (NIH), National Science Foundation (NSF), United States Department of Agriculture (USDA), Department of Defense (DOD), United States Agency for International Development (USAID), and the Centers for Disease Control and Prevention (CDC)], international organizations [World Health Organization (WHO), Pan American Health Organization (PAHO), and Food and Agriculture Organization of the United Nations (FAO)], and foreign governments. In 1992, Shope was a co-editor of the Institute of Medicine’s seminal publication, *Emerging Infections: Microbial Threats to Health in the United States*, which highlighted the threat that emerging infections pose for the U.S. and global public health [15]. Shope was also an early proponent of the effect of ecological and global climate changes on arboviral and other vector-borne diseases [15,16], and he often served on national and international committees addressing this problem.

Consultancies, service on government committees and free diagnostic laboratory support (the old RF model) could not provide sufficient funds to maintain and recruit new personnel, upgrade equipment, or support a major research program at a university in the U.S. In turn, leaders at Yale Medical School decided that arbovirology was passé and that other areas, such as HIV/AIDS, environmental health, and molecular biology, offered better funding and research opportunities for the 1980s. Thus, YARU gradually lost university support, faculty positions, and space. In 1995, after 30 years at Yale, Shope retired. Along with his colleague Robert Tesh, he moved to the newly established Center for Tropical Diseases at the University of Texas Medical Branch in Galveston [17]. The extensive WRCA virus and reagent collections went with them. Their departure and the loss of the WRCA collections was soon followed by the departure of other, younger, faculty associates, resulting in the eventual demise of YARU as a center for arbovirus research and discovery.

### 2.2. Pasteur Institutes and the French Biomedical Research Network in West Africa

Since the establishment of the Institut Pasteur in Paris in 1888, Louis Pasteur sent collaborators to various countries, mainly in the French colonies of Indochina and Africa. In that early time, Pasteur wanted to set up rabies centers where the disease was highly and dramatically prevalent; naturally, the research potential of these centers was rapidly extended to tropical infectious diseases [18]. Currently, spread among 25 countries on five continents, there are 22 institutions, 19 of which bear the name of “Institut Pasteur” (IP). Altogether, these institutions constitute a structure long called the "Institut Pasteur d’Outre-mer", which in 1988 became the International Network of Pasteur Institutes (Réseau International de l’Institut Pasteur, RIIP) and Associated Institutes. From this emerging network, the first African laboratory for microbiology was created in Saint Louis, Senegal in 1896, and then transferred to Dakar to become the Pasteur Institute of Dakar (Institut Pasteur de Dakar, IPD).

Another French multidisciplinary worldwide institution, the French Institute of Research for Development (Office de la Recherche Scientifique et Technique Outre-Mer, ORSTOM) joined the RIIP’s efforts in the intertropical zone of Africa in its fight against infectious diseases, by bringing expertise on medical entomology and vector transmitted diseases. ORSTOM was created in 1946 from a previously existing Office of Colonial Research (Office de la Recherche Scientifique Coloniale, ORSC), established by Charles de Gaulle in 1943.

Yellow fever was to play a key role in the research at IPD. In the 1930s, IPD had developed a vaccine against yellow fever (strain FNV) and produced it commercially. The re-emergence of yellow fever in Africa in the 1960s occurred mainly in the savannah zone and revealed the lack of knowledge about the natural maintenance of the virus during the inter-epidemic period. A large study to understand the emergence and re-emergence of sylvatic yellow fever was established between the IPD, Institute Pasteur Abidjan (IPA) in Côte d’Ivoire, and Institute Pasteur Bangui (IPB) in the Central African Republic, in order to detect the circulation of the YFV and map its emergence in the different ecozones. Permanent research stations were developed in rain forests and savannas to detect virus circulation in vectors and hosts from the tree canopies to the savanna ground. Year-round mosquito and monkey sampling over a period of more than 10 years made possible the detection of YFV in various monkey and mosquito species (e.g., *Aedes africanus*, *Aedes opok*, *Aedes furcifer-taylori,* and *Aedes luteocephalus*), thus elucidating the mechanism of YFV maintenance in nature. Epidemiological observations during this period allowed Max Germain to formulate the concept of a “yellow fever zone of emergence” in West and Central Africa [19]. These ecological transition zones constitute ecotones adjacent to sylvatic environments, where prevailing ecological conditions, such as vector abundance, presence of non-human primates, and closeness for human contact, enable the cross-species transmission of viruses into humans—a “zone of emergence”, which clearly appeared as the main source of epidemics in West Africa [20]. Thus, this specific ecosystem constitutes an ideal transition ecotype, where vaccination campaigns for the containment of epizootics and ultimately eradication ought to concentrate [21]. Lastly, virus isolation in male mosquitoes (*Ae. furcifer-taylori*) allowed the documentation of vertical transmission, allowing for virus maintenance in the inter-epidemic periods [22].

In the early 1960s, research laboratories focusing broadly on arboviruses were established under the umbrella of the Collaborating Center for Reference and Research on Arboviruses (CRORA), led by Paul Brés. CRORA laboratories were created at various IPs throughout Africa, including IPD, IPA, and IPB, as well as IP Yaoundé (Cameroon), and IP Tananarive (Madagascar). One of the major activities was to establish an inventory of arboviruses circulating in various ecosystems. Although virus isolations were made at various IPs, virus characterization and identification were carried out at the CRORA reference center at IPD, followed by confirmation at YARU, with final registration in the International Arbovirus Catalogue [23].

At the end of the 1970s, following an outbreak of the Ebola epidemic in Zaire in 1976, IPB set up a research program on viral hemorrhagic fevers that lasted until 1983 [24]. This research program was the result of an important collaboration between the various Pasteur Institutes in Africa and researchers of ORSTOM. In 1977, ORSTOM extended its field of research and expertise to tropical medical virology, thus supporting teams from the RIIP. The partnership between RIIP and OSTROM was instrumental in expanding the scope of field research in Africa, with seminal studies on the vertical transmission of arboviruses in mosquitoes [25] or the role of environmental factors, such as climate and latitude, in arbovirus transmission from arthropod to vertebrate hosts, using YFV [26,27] and dengue virus (DENV) [28,29,30] as models.

**Figure 1:**
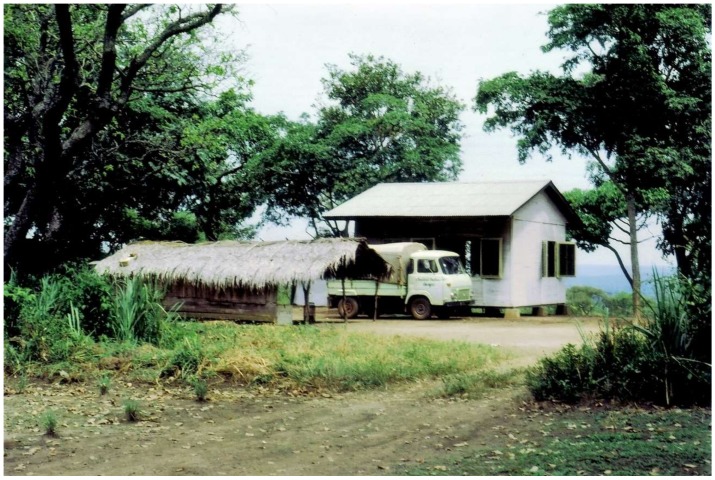
The field station in Bozo, Central African Republic.

Research at IPB was also critical in elucidating the etiology of exanthematous fevers, coined “Congolese red fevers”, that have long been attributed to rickettsia. A total of 16 arboviruses (chikungunya, Igbo-Ora, O’Nyong Nyong, Sindbis, Bouboui, yellow fever, Wesselsbron, West Nile, Zika, Ilesha, Bwamba, Dugbe, Tataguine, Nyando, Bangui, and Rift Valley fever viruses) were associated with these syndromes. Symptoms, consisting of fever, diffuse pain, and exanthem, were present in more than 60% of the cases, with etiology dominated by four viruses—Chikungunya, Ilesha, Bwamba, and Tataguine [31,32]. Between 1976 and 1986, two field research stations were established in the Central African Republic, one located adjacent to the forest (Bouboui) and the other in the wooded savanna (Bozo) (Figure 1). During this period, more than 420,000 mosquitoes of identified species in 14,591 pools were preserved for virus isolation. The most common anthropophilic mosquitoes caught were *Aedes (stegomiya) africanus* and *Ae. (st.) opok,* inside the forest gallery, *Aedes vittatus*, in the savannah, and *Anopheles gambiae* and *An. Funestus*, in the houses of the village of Bozo. A total of 321 viruses were isolated and assigned to 24 different species. These included chikungunya, Bagaza, Bouboui, Bozo, Bwamba, Ilesha, Kamese, Kedougou, Middleburg, Mossuril, M’Poko, Nyando, Orungo, Pata, Pongola, Simbu, Sindbis, Tataguine, Wesselsbron, West Nile, yellow fever, and Zika viruses. Altogether, six arboviruses were found in the forest gallery, including Bouboui, Bozo, chikungunya, Orungo, yellow fever, and Zika viruses, vectored primarily by *Ae. africanus*.

Research at IPD and IPB was also instrumental in demonstrating the expanded range of the Crimean–Congo hemorrhagic fever virus (CCHV) in West and Central Africa [33,34,35], followed by repeated isolation of the virus. This allowed a clear understanding of its eco-epidemiology in the region, including north–south migration of the infected ticks through the cattle traffic migration patterns in the Sahel [34,36]. Likewise, active circulation of the Rift Valley fever virus (RVFV) was also demonstrated in Senegal [37], Mauritania [35], Upper Volta (present day Burkina Faso) [35,38], and the Central African Republic [39].

Support for RIIP laboratories located in Africa was provided by the IPP and OSTROM teams, as well as through collaboration with various research centers in the U.S., such as Harvard University, supporting the initial study on yellow fever and FNV vaccine; YARU, as a partner for new arbovirus classification; the Centers for Diseases control (CDC) at Fort Collins Colorado, for the study of arboviruses; the CDC in Atlanta and the U.S. Army Medical Research Institute of Infectious Diseases (USAMRIID), for the initiation of viral hemorrhagic fever research in Central and West Africa.

### 2.3. Australia

The era of virus discovery in Australia can be traced to the summer of 1950–1951, when a major epidemic of encephalitis swept through southeastern Australia. There were clinical cases reported in Victoria, New South Wales, and South Australia, of which 19 (42%) were fatal [40]. A similar epidemic of unknown etiology (named Australian X disease) had occurred in eastern Australia from 1916 until 1925, with almost 300 reported cases and an average case/fatality rate of 68% [40,41]. Surprisingly, no further cases were reported in the intervening 25 years. Amongst those investigating the 1950–1951 epidemic were John A.R. Miles and colleagues at the Institute of Medical and Veterinary Science in Adelaide, and Eric L. French of the Walter and Elisa Hall Institute of Medical Research in Melbourne who, almost simultaneously, reported the isolation of a virus from the brain tissue of clinical cases [42,43]. The virus, named Murray Valley encephalitis virus (MVEV), was shown to be closely antigenically related to Japanese encephalitis virus (JEV), a flavivirus (then designated group B arbovirus) known to cause fatal encephalitis in East and Southeast Asia [43].

**Figure 2:**
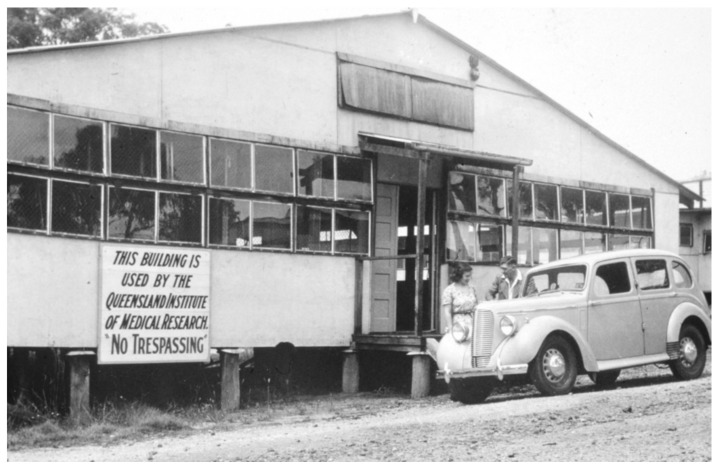
The building in Brisbane that was occupied by the Queensland Institute of Medical Research (QIMR) from 1947 until 1979 (provided with permission from QIMR).

The subsequent development of arbovirology and the pathway to virus discovery in Australia were linked intimately with the 1947 establishment of the Queensland Institute of Medical Research (now the QIMR-Berghofer Institute) at Herston in Brisbane (Figure 2). Ralph L. Doherty joined the staff and, in 1957, established a program of virus isolation from mosquitoes, based at a field station at Innisfail in the far north Queensland. In 1959, Doherty isolated the Ross River virus (RRV) from *Aedes vigilax* mosquitoes collected in Townsville [44]. He subsequently showed that RRV neutralizing antibodies occurred commonly in human sera in eastern Australia [45]. He also showed that individuals suffering from a severe debilitating syndrome, known as epidemic polyarthritis, had high antibody titres to RRV, suggesting a causal relationship [46,47]. In 1971, Doherty and his colleagues isolated the virus from a boy from the Edward River Mission aboriginal settlement in Cape York [48]. RRV and the related alphavirus, the Barmah Forest virus (see below), are now recognized as important public health problems in much of Australia and the Pacific Islands, causing arthritis, myalgia, and fatigue for six months or longer. Several thousand cases of the disease are notified annually [49].

Doherty’s program of virus discovery continued until 1977 with a team that included several other notable scientists, including Harry A. Standfast, Brian H. Kay, Edwin G. Westaway, Barry M. Gorman, and John G. Aaskov. During 1960 and 1961, Doherty isolated 60 strains of 11 viruses from 25,901 mosquitoes of 32 species from Kowanyama (then the Mitchell River Mission aboriginal settlement), Normanton, and Cairns in far north Queensland. These included four novel flaviviruses (Kunjin, Kokobera, Edge Hill, and Stratford), three orthobunyaviruses (Koongol, Wongal, and Maputta) and one orbivirus (Corriparta) [45]. The study also identified two alphaviruses previously unknown in Australia (Sindbis and Getah) and the first isolations of MVEV from mosquitoes [45]. With support from the Rockefeller Foundation, a field station was established at Kowanyama and further expeditions were undertaken to collect arthropods and potential mammalian hosts throughout Queensland. Anopheline mosquitoes and a swamp pheasant (*Centropus phasianinus*) collected at Kowanyama from 1963 to 1966 yielded three novel viruses (Kowanyama, Trubanaman, and Alfuy viruses) [50]. In 1969–1970, three novel viruses were isolated at Kowanyama (Wongorr and Mitchell River viruses from mosquitoes and the Ngaingan virus from biting midges), three novel viruses were isolated from mosquitoes collected near Charleville in western Queensland (Charleville, Warrego, and Wallal viruses) and two viruses (Belmont and D’Aguilar viruses) were isolated from mosquitoes and biting midges, respectively, collected near Brisbane [51,52]. Further expeditions to Charleville to collect mosquitoes resulted in the isolation of two novel viruses in 1974 (Facey’s Paddock and Murweh viruses) and two novel viruses in 1976 (Parker’s Farm and Little Sussex viruses) [53]. Leanyer virus was also isolated in 1974 from mosquitoes collected near Darwin in the Northern Territory [54]. Viruses were also isolated from wildlife hosts; the Almpiwar virus was isolated from a skink (*Cryptoblepharus virgatus*) at Kowanyama in 1966 [55] and the Mossman virus was first isolated from a rodent (*Rattus leucopus*) captured near Mossman in 1970 [56].

In collaboration with Doherty and his QIMR team, expeditions were also conducted to isolate viruses from ticks associated with sea birds. In 1966, Harald N. Johnson from YARU collected soft ticks (*Ornithodoros capensis*) from sooty tern (*Onychoprion fuscatus*) colonies on the Great Barrier Reef near Cairns, from which two viruses (Upolu and Johnston Atoll viruses) were isolated [57]. The Saumarez Reef virus was subsequently isolated by Toby D. St. George and colleagues from Australia’s Commonwealth Scientific and Industrial Research Organization (CSIRO) in 1974, from the same species of ticks associated with sooty terns on a coral cay in the southern Coral Sea [58]. In 1972, M. Durno Murray from CSIRO undertook an expedition to the Australian territory of Macquarie Island in the Southern Ocean to collect hard ticks (*Ixodes uriae*) associated with sea birds, resulting in the isolation of two novel viruses (Nugget and Taggert viruses) [59]. A second CSIRO expedition to Macquarie Island in 1975 yielded two additional novel viruses (Gadget’s Gully and Precarious Point viruses) from hard ticks collected in royal penguin (*Eudyptes chrysolophus schlegeli*) rookeries [60].

Other research groups in Australia also joined the hunt for arboviruses during the late 1960s and early 1970s, including Ian D. Marshall at the Australian National University in Canberra and Neville F. Stanley at the University of Western Australia. From 1965 to 1975, Marshall and his colleagues conducted surveys for arbovirus activity, particularly RRV and MVEV, in coastal regions of New South Wales and in the Murray River Valley. In addition to these and other known arboviruses, Marshall and colleagues isolated several novel arboviruses from mosquitoes, including Gan Gan, Yacaaba, Tilligerry and Termeil, Paroo River, Picola, and Barmah Forest viruses [61,62] and a novel reovirus, Nelson Bay virus, from a fruit bat (*Pteropus poliocephalus*) [63]. Like the related alphavirus RRV, the Barmah Forest virus was subsequently shown to be a cause of epidemic polyarthritis in humans [64,65], with infections occurring commonly throughout Australia [66]. The Gan Gan virus also infects humans and is suspected of an association with epidemic polyarthritis [67]. Marshall also conducted a number of collecting trips to Papua New Guinea from 1965 to 1975 funded by the Rockefeller Foundation. During an expedition to the Sepik River District of Papua New Guinea in 1965–1966, he isolated the Joinjakaka virus from a mixed pool of culicine mosquitoes and the Japanaut virus from both culicine mosquitoes and a fruit bat (*Syconycteris crassa*). In Western Australia, Stanley and colleagues surveyed for arbovirus activity in the Ord River Valley from 1972 to 1976 [68,69]. From 52,000 mosquitoes of 20 species, 195 virus isolates were recovered, including 28 isolates of MVEV and 20 isolates of the Kunjin virus from *Cx. annulirostris*, suggesting the region may be an endemic focus in Australia [69]. The study also identified eight novel viruses, including Kimberly, Parry’s Creek, Ord River, and Kunnanurra viruses, as well as four unknown isolates (OR379, OR512, OR869, and OR540), which have yet to be characterized [69,70]. Continuing surveillance in Western Australia by others has continued to reveal novel arboviruses, including Oak Vale, Stretch Lagoon, Parry’s Lagoon, and Fitzroy River viruses [71,72,73,74].

In 1968, CSIRO established a new virology laboratory at Long Pocket in Brisbane, headed by Toby D. St. George, to investigate endemic diseases of livestock in northern Australia. In 1967, Doherty and colleagues had isolated bovine ephemeral fever virus (BEFV) from cattle during a major epizootic in Queensland [75] but, despite epidemiological evidence suggesting vector-borne transmission, the virus had never been isolated from insects. This led St. George and colleagues to attempt virus isolations from a location in northern Australia, where serological monitoring of a sentinel herd of cattle indicated that BEFV was likely to be enzootic. For a continuous period from October 1974 until May 1976, insect collections for virus isolation were conducted at Beatrice Hill southeast of Darwin. From the 57,596 mosquitoes and 175,880 biting midges processed, one isolate of BEFV was recovered (from a mosquito pool). However, the collection also yielded 93 other virus isolates from 22 different serological groups, including four novel viruses (CSIRO Village, Marrakai, Beatrice Hill, and Humpty Doo virus) [76]. Most significantly, the collection also yielded a single isolate of a novel serotype of bluetongue virus (BTV serotype 20, BTV-20), a major pathogen of sheep and goats that had previously been regarded as exotic to Australia [77].

The isolation of BTV-20 and the consequences for international trade dramatically changed the landscape with respect to virus discovery and characterization in Australia. An immediate consequence was the approval of government expenditure for the establishment of the $230 million CSIRO Australian Animal Health Laboratory (AAHL) in Geelong, Victoria, providing high-level biosecure containment for laboratory work and live animal studies. The discovery also led to the establishment of a permanent veterinary virology capability at the Berrimah Veterinary Laboratory in Darwin under Geoff P. Gard, and a national program for serological monitoring of sentinel cattle herds and the collection of insect vectors. Efforts to isolate viruses from arthropods and livestock intensified. In the Northern Territory, a second novel serotype of bluetongue virus (BTV-21) was isolated from a healthy sentinel cow at Victoria River Station in 1979 [78], and four other novel arboviruses (Coastal Plains, Berrimah, Adelaide River, and Koolpinyah viruses) were isolated from healthy cattle between 1981 and 1985 [79,80,81,82]. In Queensland, eight novel arboviruses were first isolated between 1976 and 1981 from biting midges (Tibrogargan, Tinaroo, Peaton, Wongabel, and Walkabout Creek viruses), healthy sentinel cattle (the Douglas virus), and soft ticks (*Argas robertsi*) (Vinegar Hill and Lake Clarendon viruses) [55,83,84,85,86,87]. Surveillance activities by the Berrimah Veterinary Laboratory have continued to the present, with regular reports of the isolation of novel arboviruses. Continuing surveillance by others in northern Australia has also resulted in the isolation from mosquitoes of the Bamaga virus and New Mapoon virus from Cape York [88,89].

The 1990s also saw several significant disease emergence events in Australia, which drew particular attention to bats as reservoir hosts of highly pathogenic viruses. In September 1994, an outbreak of a severe respiratory disease occurred in horses at a stable in Brisbane. Of the 21 affected horses 14 were euthanized or died of the disease. One of two severely affected humans who had contact with the horses also died. Cooperation between the Queensland Government, the newly established CSIRO Australian Animal Health Laboratory, and others resulted in rapid isolation of the Hendra virus, a novel paramyxovirus [90], and the identification of fruit bats as reservoir hosts [91,92]. The Hendra virus has since re-emerged regularly in Australia, with more than 70 confirmed cases in horses and seven infected humans, four of whom have died. In 1996, an injured female fruit bat (*Pteropus alecto*) was found at Ballina in New South Wales. Tissue homogenates from the euthanized bat were injected into mice, resulting in the isolation of a novel lyssavirus, subsequently named Australian bat lyssavirus (ABLV) [93]. Three fatal human cases of ABLV infection have subsequently been reported [94,95,96]. The virus is now known to occur at low prevalence in five of six families of bats endemic to the Northern Territory, Queensland, and Western Australia [97]. In April 1997, another novel paramyxovirus, the Menangle virus, emerged at a commercial piggery in New South Wales, causing stillbirths with abnormalities of the brain, spinal cord, and skeleton [98]. Two humans exposed to the pigs also developed an influenza-like illness [99]. Fruit bats were again implicated as reservoir hosts [100]. The role of bats in the ecology and emergence of pathogenic viruses has been a major focus of study in Australia since that time, primarily involving research teams led by Hume E. Filed and Linfa Wang.

In all, more than 80 novel RNA viruses representing 9 families and 16 genera have been isolated from humans, livestock, wildlife, and arthropods in Australia and Papua New Guinea, and reported in the literature (Table S1). Many others have been isolated but remain uncharacterized and/or unreported. More complete characterization of these viruses will be facilitated greatly by the use of NGS.

### 2.4. South and Southeast Asia

South and Southeast Asia have also been a fertile area for virus discovery, particularly novel mosquito-borne and tick-borne flaviviruses and viruses with reservoirs in bat species. Early studies in India, partly funded by the Rockefeller Foundation, by Telford Work and his Indian colleagues from the Virus Research Centre in Pune, including D.P. Murthy, P.N. Bhatt, H. Trapido and K. Pavri, led to the discovery of the Kyasanur Forest disease (KFD) virus [101]. The virus was isolated from sera and tissues collected from a moribund black-faced langur (*Presbytis entellus*). This followed reports of an epizootic of unknown etiology causing large numbers of deaths in non-human primates and a number of cases of severe febrile illness in villages close to forested areas where dead monkeys had been found. The virus was shown to be closely related to the Russian spring–summer encephalitis (RSSE) serocomplex of flaviviruses, now known as the tick-borne encephalitis (TBE) serocomplex of flaviviruses. The virus was also isolated from some larvae and nymphs of *Hemaphysalis spinigera* ticks [102]. KFD in humans followed a biphasic course, not unlike TBE, but with some hemorrhagic manifestations not seen in TBE, and without either meningitis or encephalitis. Another tick-borne virus related to the RSSE serocomplex, Langat virus, had been isolated two years earlier from a pool of hard ticks, *Ixodes granualtus*, collected from forest rats caught near Kuala Lumpur, Malaysia, by C.E. Gordon Smith, then working at the Institute for Medical Research in Kuala Lumpur [103], but it is not known to be a human pathogen.

A number of mosquito-borne flaviviruses were first isolated in Southeast Asia. The most important with respect to human disease are three of the four dengue serotypes. Although dengue serotype 1 had been first isolated independently by Hotta in Japan in 1943 [104], and shortly after by Sabin in Cincinnati in 1945 with material collected in Hawaii [105], the other three serotypes were first isolated from material collected in Southeast Asia. Dengue serotype 2 was also isolated by Sabin in 1945 from material obtained from New Guinea [105] and dengue serotypes 3 and 4 were first isolated in 1956 from human sera and *Aedes aegypti* and *Culex tritaeniorhynchus* mosquitoes collected during a major outbreak of epidemic hemorrhagic fever in Manila, Philippines, by W.M. Hammon, A. Rudnick, and colleagues at the University of Pittsburgh [106]. Other novel flaviviruses have been isolated in Malaysia, Thailand, and Papua New Guinea [107]. The Tembusu (TMUV) virus was isolated in Kuala Lumpur in 1957 from various mosquito species [108] and was the first of several closely related viruses, including the ThCAr virus, which was isolated from a pool of *Cx. tritaeniorhynchus* mosquitoes collected in Chiang Mai, Thailand, in 1992 [109]; the Sitiawan virus, from sick broiler chicks in Malaysia [110]; and the duck Tembusu virus, an infection of ducks and geese causing an egg-drop syndrome in China and Southeast Asia [111,112]. Neutralising antibodies were found in humans in Malaysia [113] but the virus has not been implicated in human disease. Two other mosquito-borne flaviviruses have been described, Jugra virus and Sepik virus. Little is known about the Jugra virus, which was isolated from *Aedes* spp. and *Uranotaenaia* spp. mosquitoes and from the blood of a *Cynopterus brachyotis* fruit bat [108]. The Sepik virus was isolated in 1966 by Ian D. Marshall and colleagues from a pool of *Mansonia septempunctata* mosquitoes collected in the Sepik District of Papua New Guinea [114]. It was associated with a hospitalized case of febrile illness of unknown origin, with rising neutralising antibody to the virus. The Sepik virus is particularly interesting, as its nucleotide sequence analysis shows it to be the closest known flavivirus to yellow fever virus [115]. A novel lineage of the West Nile virus was isolated in Sarawak, East Malaysia, by D.I.H. Simpson, E.T.W. Bowen, and colleagues, from *Cx. pseudovishnui* group mosquitoes [116]. Initially called Kunjin virus, it was shown to differ significantly in genomic sequence from the Australian Kunjin viruses, which have been shown to comprise West Nile lineage 1b viruses, and have been described as West Nile lineage 6 virus [107].

Two flaviviruses with no known vector have been isolated in Southeast Asia, both from *Cy. brachyotis* fruit bats: the Carey Island virus was isolated from a bat in the Jugra Forest, Malaysia, in 1970 by A. Rudnick and colleagues from the Institute of Medical Research, Kuala Lumpur and the International Center for Medical Research, University of California [108], and the Phnom Penh virus was isolated by J.J. Salaun and colleagues in 1969 from the salivary glands and brown of bats [117]. A closely related virus, the Batu Cave virus, is considered to be a variant of Phnom Penh virus.

A considerable number of novel bunyaviruses have been isolated from South and Southeast Asia, particularly by scientists from the National Institute of Virology (formerly the Virus Research Centre) in Pune, including P.N. Bhatt, K. Pavri, K.R. Singh, C.N. Dandawate, F.M. Rodrigues, D.T. Mourya, P.D. Yadev, A.C. Mishra, and many others. Recently reviewed in [118], these viruses include the orthobunyaviruses, Umbre, Kaikalur, Thimiri, and Sathuperi viruses; a nairovirus, Ganjam virus; phleboviruses, Bhanja, and Malsoor viruses; a hantavirus, Thottapalayam virus; and two uncharacterized viruses, Kaisodi and Wanowrie viruses. Additionally, novel bunyaviruses, Batai and Oya viruses, have been isolated in Malaysia, the former from *Cx. gelidus* mosquitoes [108] and the latter from pigs [119], and the Kaeng Khoi virus was isolated from *Tadarida plicata* bats in Thailand.

Novel orbiviruses from South and Southeast Asia include the Sathuvachari virus, isolated from starlings (*Brahminy myna*) collected in Vellore, Tamil Nadu, India, and most closely related to the mosquito-borne orbiviruses [120]; and the Japanaut virus, isolated from a mixed pool of culicine mosquitoes from Papua New Guinea [108].

New rhabdoviruses first isolated in South Asia include the Chandipura virus and Joinjakaka virus—The former, a major human pathogen in India, was isolated from a human infection in 1965 near Nagpur City [121], whereas the latter, isolated in 1966 from a mixed culicine pool in the Sepik District of Papua New Guinea, is not associated with disease in humans or animals. Chandipura infection is characterized by fever, chills, arthralgia, myalgia, vomiting, and weakness.

Two novel alphaviruses were reported in Kuala Lumpur—Bebaru and Getah viruses. Bebaru was first isolated from *Cx. (Lophoceraomyia)* spp. collected in 1956, but although neutralising antibodies have been found in human sera, it has not been associated with human disease [108]. Getah virus was first isolated from *Cx. gelidus* mosquitoes collected in 1955 near Kuala Lumpur [108]. It causes a mild disease in horses, characterized by pyrexia, edema of the hind limbs, swelling of the submandibular lymph nodes, and urticarial rash. It also causes a mild disease in pigs, with occasional reproductive problems, including abortion and neonatal infections. Neutralising antibodies have been found in a number of animals and in humans.

The important role of bats as reservoirs of a wide range of viruses was underlined by a number of the viruses described above, and particularly by the discovery of their role as the reservoir of the Nipah virus in Malaysia in 1999 [122] and subsequently their probable role as the origin of severe acute respiratory syndrome coronavirus (SARS-CoV) [123,124]. The Nipah virus, a virus closely related to the Hendra virus in Australia, was first isolated K.B. Chua and S.K. Lam during an outbreak of severe disease of humans and pigs in 1998–1999 in Peninsula Malaysia [125], resulting in 265 human cases with a mortality of 40%, and the culling of over 1 million pigs. Transmission to humans was from infected pigs. The disease in humans was a rapidly progressive encephalitic syndrome, with a significant pulmonary syndrome in some patients [126]. In pigs, the disease was spread via the respiratory tract, and the symptoms were either neural or pulmonary, or both. Subsequent epidemics in Bangladesh and India have substantially expanded our knowledge of the Nipah virus, and have demonstrated that direct transmission of the virus from bats to humans can occur through the consumption of date palm juice, and possibly by other routes, and that mortality rates may often be significantly greater than in Malaysia [127]. Furthermore, evidence of Nipah-like and Hendra-like viruses have been detected either by isolation or serology from other pteropid bats across the geographic range of the genus, and related viruses may be carried by other bat species on other continents. SARS-CoV was first isolated by M. Peiris and colleagues in Hong Kong [128], and contemporaneously in the U.S. [129] and Europe [130]. It was shown to be unrelated to other coronaviruses. Transmission to humans is believed to have been via an intermediate host, such as the Himalayan palm civets (*Paguna larvata*), through wet markets in southern China.

Continued studies of the Nipah virus in Malaysia, and subsequently in Bangladesh and India, and investigations of SARS-CoV in bats have resulted in an enormous explosion of knowledge of viruses carried by bats, with many examples from most viral families, although in many cases the information is from genomic fragments [131]. Virus isolations have been made from bats, especially frugivorous bats, from India and Malaysia. One of the earliest isolations was a paramyxovirus of the genus *Rubulavirus*, which was isolated from a *Rousettus leschenaultia* bat collected near Pune in India [132]. A novel adenovirus from the genus *Mastadenovirus* was also isolated from the same fruit bat species caught in Maharashtra State [133]. A number of viruses have been isolated from fruit bats in Malaysia by K.B. Chua, S.K. Lam, L.F. Wang, and their colleagues—Tioman virus, a paramyxovirus in the genus *Rubulavirusi*, isolated from *Pteropus hypermelanus* and related to the Australian Menangle virus, but not known to cause human disease [134]; Pulau virus, an orthoreovirus related to the Nelson Bay virus of Australia, and not associated with human or animal disease [135]; Melaka virus, an orthoreovirus, causing acute respiratory disease in humans [136]; and Kampar virus, an orthoreovirus related to the Melaka virus, and also causing acute respiratory disease [137]. The importance of orthoreoviruses originating in pteropid bats was assessed in an outpatient clinic, where it was found that pteropine orthoreoviruses are among one of the common causative agents of acute upper respiratory tract infection (URTI), with a cough and sore throat as the most common presenting clinical features [138].

### 2.5. USSR/Russia

The history of arbovirus research in the USSR began in 1937, when an expedition under the leadership of Lev A. Zilber (at the time, the head of Central Virological Laboratory of Narkomzdrav USSR in Moscow) went to the Russian Far East to study seasonal epidemic encephalitis. This disease with high mortality rates affected forest workers and soldiers stationed in the taiga, mainly those who came from other regions of the USSR. The disease had a pronounced seasonality—the cases started being recorded at the beginning of May, reached the peak in early June, and declined by August. Local doctors designated the disease “spring–summer encephalitis” (SSE) and assumed that it had been caused by some kind of virus. It has also been suggested that there were some similarities between SSE and “summer encephalitis” (Japanese B encephalitis and St. Louis encephalitis) described at the time, it was also assumed to be a toxic form of influenza [139]. However, the etiology and transmission routes of SSE remained unclear. During summer of 1937, Zilber and colleagues isolated at least 29 strains of a new virus from the blood and cerebrospinal fluid of sick people, and from brain tissues of dead patients. The isolated virus had a weak antigenic relationship (in complement fixation tests) with Japanese B encephalitis virus [140]. Based on comparative analysis of epidemiologic data and the seasonal abundance of *Ixodes persulcatus* ticks in the taiga, Zilber assumed that SSE was transmitted by ticks, in contrast to “summer encephalitis”, which is transmitted by mosquitoes [141]. Several strains of the virus were isolated from the *Ix. persulcatus* ticks, and their ability to transmit the virus by biting laboratory animals was shown experimentally [142]. One of the first isolated strains (Sofjin) was used for infecting rhesus macaques, which developed the clinical symptoms with signs of central nervous system (CNS) impairment, similar to those in sick people [141,143]. So, the etiological agent of SSE, which was subsequently given the name tick-borne encephalitis, was discovered and is now known by the name tick-borne encephalitis virus (TBEV). In 1938–1939, subsequent expeditions under the leadership of Pavlovsky and Smorodintsev studied in detail various aspects of the ecology, epidemiology, and pathogenesis of TBEV, as well as the protective properties of the first anti-TBE vaccine, obtained from the brain tissue of mice infected by the TBEV strain Sofjin [144,145,146]. Further studies showed that TBEV is also prevalent in other regions of the USSR, including the European part, where the main vector of the virus is *Ix. ricinus* ticks. At the same time, it was found that TBEV is also an etiological agent of some seasonal encephalitis or febrile illnesses, such as Central European encephalitis or biphasic milk fever [147,148,149]. The strains of TBEV were initially divided into two geographical subtypes (“Far Eastern” and “Central European”). These subtypes differed in severity of the illness and had antigenic differences in virus neutralization tests with serum of convalescents [148]. A third subtype of TBEV (“West Siberian”) was described by Pogodina and her colleagues in 1981 [150]. Genetic data that has been accumulating since the late 1980s confirms the existence of three main TBEV subtypes (or genotypes). The nucleotide difference between genotypes reaches 15–20% when comparing complete genomes [151,152,153].

TBE is the most important arboviral infection in Russia. Despite significant progress in the development of anti-TBEV vaccines, thousands of cases are recorded annually in Russia, mainly in Siberian and Far Eastern regions [154]. In the modern classification, TBEV belongs to the species *Tick-borne encephalitis virus* of the genus *Flavivirus* (*Flaviviridae*) [155]. TBEV is widely distributed within the area of its main arthropod vector—*Ix. persulcatus and Ix. ricinus* ticks, including Russia, Eastern and Central Europe, Baltic and Scandinavian countries [156,157].

The discovery of TBEV as a causative agent of SSE gave an impetus to studies of similar diseases throughout the USSR. During subsequent years, the major virological centres were established as parts of the Academy of Medical Science of the USSR (AMS USSR), such as the department of neurovirology at the Institute of Neurology (1942), the Institute of Virology (1944), the Institute of Poliomyelitis and Viral Encephalitis (1950), as well as departments of virology at regional medical institutes in Siberia and the Far East. Scientists from these centres were actively involved in the study of various zoonotic viral infections distributed in the USSR. Many participants of the first expeditions subsequently became famous virologists. One of the most notable ones is Michael P. Chumakov, who later headed the Institute of Virology AMS USSR (1950–1954) and the Institute of Poliomyelitis and Viral Encephalitis AMS USSR (1955–1972) in Moscow. Chumakov organized numerous expeditions that aimed to study the etiology of zoonotic human infections. In the 1940s, outbreaks of the disease, designated by local doctors as “atypical tularemia”, “anicteric leptospirosis”, and “Omsk spring–summer fever”, were recorded in several rural regions of the Omsk district in Western Siberia. Clinicians from the Omsk Medical Institute, under the leadership of Ahrem-Akhremovich, described the disease in detail and named it Omsk hemorrhagic fever (OHF), as the patients often developed hemorrhagic diathesis. They also suggested that OHF was transmitted by *Dermacentor reticulates* ticks, which are highly prevalent in the region [158,159]. In 1947, Chumakov and colleagues investigated the blood of patients with OHF and isolated 40 strains of a new virus, which was similar but different from TBEV in serologic tests. The virus was named Omsk hemorrhagic fever virus (OHFV). Several strains of OHFV were also isolated from *De. reticulates* ticks collected in the natural foci of OHF [160,161]. In subsequent years, the ecology of OHFV was extensively studied by scientists from the Omsk Medical Institute and the Institute of Poliomyelitis and Viral Encephalitis AMS USSR. The *De. reticulatus* ticks and their host, a narrow-headed vole (*Microtus gregalis*), are considered an original natural reservoir of OHFV. The European water vole (*Arvicola amphibius*), the tundra vole (*Microtus oeconomus*), and some species of shrews are also involved in the circulation of OHFV [156]. However, the emergence of OHF outbreaks in the 1940s was presumably a consequence of the introduction by humans of muskrats (*Ondatra zibethicus*) to this region in 1935–1936 [162]. Muskrats are highly susceptible to OHFV and serve as an extremely effective amplifying host. The appearance and growth of the muskrat population in the natural foci of OHF led to an increase in infection rates in other animals and ticks [162,163]. In addition to transmission of OHFV by ticks, humans can get infected while hunting and skinning, by direct contact with blood and excretions of infected animals. Such “muskrat outbreaks” among hunters and their family members have been registered in the region at different times of the year, including winter, which is the season of active hunting for muskrats [164,165]. Based on antigenic relationships, OHFV was assigned to the TBE antigenic complex [166], and later was classified as a separate species, *Omsk hemorrhagic fever virus* of the genus F*lavivirus* (family *Flaviviridae*) [155]. Genome sequence analysis confirmed the close evolutionary relationships of OHFV with TBEV [167,168,169].

In 1944, virologists led by Chumakov studied the etiology of an outbreak of a febrile illness which was accompanied with hemorrhagic manifestations (“acute infectious capillary toxicoses”) in a rural area in the northwest part of the Crimean Peninsula. They designated the disease as Crimean hemorrhagic fever (CHF) and suggested that is transmitted by *Hyalomma (plumbeum) marginatum* ticks. Despite the absence of virus isolates from specimens from CHF patients or from ticks, the viral etiology of CHF and its zoonotic nature were proven experimentally by infecting volunteers with the blood of CHF patients or a filtered suspension of ticks collected from a hare caught in the focus of the disease [170]. Sporadic cases and outbreaks of CHF were subsequently recorded almost annually in southern regions of the European USSR and Central Asian Soviet republics. The first strains of the CHF virus were isolated by Alexander M. Butenko from Chumakov’s team in 1967, from sera of CHF patients and from *Hy. marginatum* nymphs isolated in southern Russia [171,172]. Later, the CHF virus was shown to be identical to the Congo virus isolated from a patient with hemorrhagic fever in Zaire (present day Democratic Republic of Congo) and the virus received its present name, the Crimean–Congo hemorrhagic fever virus (CCHFV) [173]. CCHFV is a one of the prototypic nairoviruses and today is assigned to the species *Crimean–Congo hemorrhagic fever virus*, of the genus *Orthonairovirus* (*Nairoviridae: Bunyavirales*).

During spring and summer 1962, Chumakov, together with Libíková from the Institute of Virology in Bratislava (former Cžechoslovakia), investigated an outbreak of fibrile illness in the Kemerovo district in western Siberia. Initially, it was assumed that the patients were affected by TBE, but the sera of the patients did not react with TBEV-specific antigen in serological tests. On the contrary, a new virus, named the Kemerovo virus (KEMV), was isolated from the blood of patients. Several strains of KEMV were also isolated from *Ix. persulcatus* ticks collected in the region where the outbreak occurred [174,175]. Similar to KEMV, the Tribeč virus and Lipovníc virus were isolated from *Ix. ricinus* ticks in Czechoslovakia in 1963 [176,177]. Based on morphological studies, KEMV was classified to the genus *Orbivirus* (family *Reoviridae*) [178]. The ecology of KEMV in Russia has not been studied sufficiently, but recent research has shown that its prevalence in *Ix. persulcatus*, *Ix. ricinus*, *Ix. Pavlovsky*, and *De. reticulatus* ticks varies from zero to 10.1% in different regions of the country [179,180].

From the above, it follows that in the period 1930–1960, arboviruses in the USSR were studied mostly as causative agents of human disease. Examinations of arthropods and vertebrates in the natural foci of important human disease often led to exploring some other arboviruses. For example, Butenko isolated the West Nile virus (WNV) and Dhori virus (DHOV) from *Hy. marginatum* ticks for the first time in the USSR while studying the natural foci of CCHFV in the southern region of Russia in 1964 [181]. In the late 1960s, there was an ecological trend in virology developing in the USSR. The founder of the ecological approach to virology in the USSR was Dmitry K. Lvov, who established the Department of the Ecology of Viruses at the D.I. Ivanovsky Institute of Virology in Moscow (1967), and later headed the Institute (1987–2014). Under his leadership, an ecological and virological survey was organized, aimed to identify the arboviral diversity in hematophagous arthropods and wild animals of the entire USSR. The survey included collecting and examining mosquitoes, ticks, and vertebrate animals (mostly rodents and birds), as well as samples from humans, in different types of biocenoses located in different climatic zones of the USSR.

Lvov and colleagues isolated more than 500 strains of different mosquito-borne viruses, including viruses of the California encephalitis antigenic group (species *California encephalitis orthobunyavirus*) and Batai and Batai-like viruses (species *Bunyamwera orthobunyavirus*) in the genus *Orthobunyavirus*, family *Peribunyaviridae* [182,183,184,185]. The other mosquito-borne viruses whose circulation was discovered and studied extensively, are the Sindbis virus (SINV) and Getah virus (GETV) (genus *Alphavirus*, family *Togaviridae*) [186,187,188].

One of the important subjects of D. Lvov’s research was *Ix. uriae* ticks, which parasitize on colony-nesting sea birds. In 1969–1974, more than 240 virus strains were isolated from *Ix. uriae* ticks collected in the nests of sea birds on the coasts and islands in the Sea of Okhotsk, the Bering Sea, and the Barents Sea [189]. The isolated strains were mostly classified as novel bunyaviruses, flaviviruses, and orbiviruses, often based on morphological studies of the virion structure only, because their antigenic relationships with other viruses were not known at the time. Among them, the Sakhalin virus (SAKHV) and Paramushir virus (PRV) were described as a novel bunyaviruses and later classified to the species *Sakhalin orthonairovirus* (genus *Orthonairovirus*, family *Nairoviridae*) [190]. Several other new viruses (Zaliv Terpenia, Comandory, and Rucutama viruses) were discovered and now belong to the species *Uukuniemi phlebovirus* (genus *Phlebovirus*, family *Phenuiviridae*) [191]. The Tyuleniy virus (TYUV) was isolated for the first time, which is one of the prototypic viruses of seabird tick-borne flaviviruses group (genus *Flavivirus,* family *Flaviviridae*) [192]. The prevalence of the Okhotsky virus (OKHV) and Aniva virus (ANIV), two newly described viruses belonging to the species *Great Island virus* (genus *Orbivirus*, family *Reoviridae*), have been studied in detail [156,189,193].

Many new viruses were discovered by Lvov and his colleagues while exploring the territories of Central Asia and Transcaucasia. They isolated and studied the Issyk-Kul virus (ISKV), which is associated with bats of the family Vespertionidae and their argasid ticks [194,195]. New viruses, Tamdy (TAMV) and Burana (BURV), were isolated from *Hyalomma* spp. ticks collected from sheep or cows in pasture lands [196]. Some novel viruses (Artashat, Chim, and Geran viruses) were isolated from argasid ticks collected in rodent burrows [197]. Morphological studies of these viruses by electron microscopy identified them as bunyaviruses. Recently, they were classified as different species of the genus *Orthonairovirus* (family *Nairoviridae*) [198].

In total, during the ecological and virological surveys of the 1970s to 2000s, thousands of strains of different arboviruses were isolated, some of which remain to be classified. Based on the studies conducted by Soviet and Russian virologists, we now know that at least 80 zoonotic viruses, assigned to eight viral families, circulate on the territories of the former USSR [156]. Does this number reflect the true diversity of viruses circulating in the vast territory of northern Eurasia? This question can only be answered by additional research aimed at finding new viruses, using new methods and approaches.

## 3. The Tools of Discovery

### 3.1. Virus Isolation and Serology

The concept of viruses developed from the observations of Ivanovsky and Beijerinck of “filterable agents”, with the discovery of the causative agent of tobacco mosaic in the 1890s. Yellow fever virus and dengue virus were the first two arboviruses to be isolated early in the 20th century [199,200]. The pioneering work of Alexis Carrel on the development of many cell and tissue culture methods in the 1910s at the Rockefeller Institute, and later refinements by Maitland, Eagle, and Enders, led to the widespread use of various culture systems as indispensable tools for virus studies [201,202,203]. Although these tools have since been used extensively for the in vitro characterization of viruses, they were inadequate for the identification and classification of a flood of novel viruses collected through the YFV surveillance program supported by the Rockefeller Foundation. Jordi Casals, among others, led the use of the complement fixation (CF) test in order to study viruses affecting the central nervous system. The CF test exploits the unique affinity of complement for antigen–antibody complexes. The original assay was developed in the 1920s for the serologic study of YFV, was improved in the 1930s [204,205], and its sensitivity and specificity improved again in the 1950s [206]. Using CF tests, Casals and his colleagues were able to classify viruses into antigenic groups. However, the inherent complexity and labour consuming aspects of the assay (titrations of antigen, complement, and hemolysin for optimal outcomes), technical demands (accurate interpretation of outcomes) and the development of alternative assays (see below), have restricted its applicability in laboratories worldwide.

Hirst observed in 1941 that chicken erythrocytes agglutinated in the presence of the influenza A virus and that virus-specific antibodies inhibited agglutination, forming the foundation for the hemagglutination inhibition (HI) test [207]. A decade later and on Theiler’s suggestion, Casals showed that many arboviruses also agglutinated erythrocytes, establishing the HI test as a diagnostic tool for arbovirus infection and identification [4]. The gold standard for arbovirus identification, the plaque reduction neutralization test (PRNT), has its origins in the observations of Stokes and colleagues, dating back in the 1920s when monkeys could be protected against YFV by the inoculation of convalescent sera from patients who had recovered from the disease [200]. By the early 1930s, Max Theiler had adapted the assay for use in mice, in which mixed serum and virus was inoculated intracerebrally [208]. The cell culture adaptation of the test was first demonstrated by Itoh and Melnick in 1957 for studying the seroconversion of Chimpanzees infected with echoviruses [209], and a year later by Henderson and Taylor to detect antibodies to the eastern equine encephalitis virus [210]. Versions of this assay are now widely used, including the microPRNT [211], the virus reduction neutralization test (VRNT) [212], the focus reduction neutralization test (FRNT) [213], the rapid fluorescent inhibition test (RFFIT) [214], the flow-cytometry neutralization [215,216], the colorimetric micro-neutralization assay (CmNt) [217], and the reporter virus particle-based neutralization assays [218,219,220,221].

Historically, these methods (virus isolation, HI, CF, and neutralization tests) served as the basis of arbovirus diagnosis for many years, augmenting electron microscopy (see section below), which allowed the visualization of viruses in infected tissues and cell cultures. However, an inherent limitation of the serology-based assays has been their inability to determine whether antibodies in the examined serum were the result of a recent or past infection. This conundrum was solved by determining whether antibodies were IgM (recent infection) or IgG (past infection), using the enzyme-linked immunosorbent assay (ELISA) [222]. The introduction of ELISA revolutionized the field by also offering increased specificity and sensitivity for the accurate detection of many viruses.

Overall, although identification of pathologic agents through serologic assays is quite straightforward, there are instances where accurate identification may not be possible due to cross-reactivity. For example, cross-reactivity among flaviviruses poses a challenge in their identification, even when the “gold standard” of PRNT for arbovirus detection is applied, especially in hyper-endemic settings of flavivirus circulation. Several diagnostic labs faced this challenge during the recent emergence and explosive spread of the Zika virus in the Americas. A similar challenge is also common for the serologic diagnosis of bunyavirus infections, which is attributed to their ability to reassort. In this scenario, a novel bunyavirus may be misidentified as a known pathogen due to the presence of the M segment (contributed by the known pathogen), which encodes the immune-reactive envelope proteins (reviewed in [223]).

### 3.2. Electron Microscopy (EM)

Since the beginning of modern virology in the 1950s, transmission electron microscopy (TEM) has been one of the most important and widely used techniques for the identification and characterization of new viruses. Two TEM techniques are usually used for this purpose: negative staining on an electron microscopic grid coated with a support film and (ultra) thin section TEM of infected cells, fixed, pelleted, dehydrated, and embedded in epoxy plastic. Negative staining can be conducted on highly concentrated suspensions of purified virus or cell culture supernatants. For some viruses, TEM can be conducted on contents of skin lesions (e.g., poxviruses and herpesviruses) or concentrated stool material (rotaviruses and noroviruses). For successful detection of viruses in ultrathin sections of infected cells, at least 70% of cells must be infected, and so either high multiplicity of infection (MOI) or rapid virus multiplication is required.

Viruses can be differentiated by their specific morphology (ultrastructure): shape, size, intracellular location or, for some viruses, from the ultrastructural cytopathology and specific structures forming in the host cell during virus replication. Usually, ultrastructural characteristics are sufficient for the identification of a virus at the level of a family. In certain cases, confirmation can be obtained by immuno-EM performed either on virus suspension before negative staining or on ultrathin sections. This requires virus-specific primary antibodies, which might be not available in the case of a novel virus. For on-section immuno-EM, OsO4 post-fixation must be omitted and the partially dehydrated sample must be embedded in a water-miscible acrylic plastic (usually LR White). The ultrastructure of most common viruses is well documented in good atlases and book chapters [224,225,226,227,228,229,230,231,232,233,234,235] and many classical publications of the 1960s, 1970s, and 1980s. Several excellent reviews were recently published on the use of TEM in the detection and identification of viruses [236,237,238].

## 4. The Advent of NGS

The advent of next generation sequencing (NGS) has expanded the tool kit of the virus hunter. For many years, Sanger-based sequence analysis has been employed in the identification and characterization of viruses [239,240,241,242]. However, with the completion of the human genome sequence, the necessity for a high throughput approach that provided a massively-parallel sequencing strategy was fully apparent [243,244]. The automated Sanger method was considered a first-generation technology and newer methods are referred to as next generation sequencing (NGS). Commercially available technologies from Roche/454, Illumina, Life Technologies/APG, Oxford Nanopore, and Pacific Biosciences offer unique NGS platforms, and all have been extensively reviewed [245,246,247,248,249]. Unlike Sanger sequencing, NGS does not require prior knowledge of the viral sequence and thus can be used for viruses of unknown sequence. Many viruses cannot be cultured in the cell culture systems currently in use. NGS has shifted the paradigm by removing the need for cell culture and so opening the door to the discovery of many new viruses. The first instruments for NGS were developed in the 1990s and commercialized in the early 2000s, and they were quickly adopted for the identification of novel viruses from a wide range of sources. The number of known viruses increased from about 3,000 in 2005 to approximately 11,000 viruses in 2013 [250], and that number has since increased dramatically. The technology has also been applied to sequence analysis of many previously known but poorly characterized viruses. This has significantly expanded the known virosphere and assisted in understanding the diversity and evolutionary relationships of viruses. The expansion of the known virosphere has also allowed the taxonomic assignment of an increasing number of viruses, with a total of 14 orders, 150 families, 1019 genera, and 5560 species of viruses currently approved by the International Committee on Taxonomy of Viruses (ICTV) [251,252,253,254,255,256]. NGS has also allowed sequence analysis of hundreds or thousands of isolates known pathogenic viruses, facilitating epidemiological studies at scales extending from very local to global. This has generated a trove of new knowledge of significant public health importance. However, without the availability of isolates, NGS has not necessarily increased our understanding of the ecology of novel viruses, their host range, and the risks they may pose to public, veterinary, agricultural, or environmental health.

### 4.1. Metagenomics and a New Era of Virus Discovery

The application of NGS to the unbiased mass sequencing and bioinformatic analysis of total nucleic acids extracted from biological samples obtained from a wide range of sources has led to an explosive increase in the number of complete or near-complete viral genomes. For example, a recent study using NGS to sequence the transcriptomes of arthropods representing 70 species from four classes (Insecta, Arachnida, Chilopoda, and Malacostraca) identified 112 novel viruses, many of which are represented by complete or near-complete genomes [257]. The novel viruses encompass the entire taxonomic diversity of previously known families and/or genera of (-) ssRNA viruses and include divergent viruses with entirely novel and unusual genome architecture. Similarly, sequence analysis of the transcriptomes of animals of more than 220 species sampled across nine metazoan phyla (Arthropoda, Annelida, Sipuncula, Mollusca, Nematoda, Platyhelminthes, Cnidaria, and Echinodermata), as well as chordates of the subphylum Tunicata (salps and sea squirts), resulted in the discovery of 1445 RNA viruses, mostly represented by complete or near-complete genomes [258]. Based on phylogenetic analysis of RNA polymerase (RdRp) domain sequences, the novel viruses included clades representing many established families of plant and animal RNA viruses, as well as at least five clades that are so divergent that they are considered as likely new virus families or orders. Also, the sequencing of transcriptomes of gut, liver, and lung or gill tissue of fish, reptiles, amphibians, and birds identified 214 novel vertebrate-associated viruses, representing every family or genus of RNA virus associated with vertebrate infection, including those containing important human pathogens (orthomyxoviruses, arenaviruses, and filoviruses) [259]. These and other similar studies have heralded a new era in virology, revealing new dimensions in viral biodiversity and providing largely unexpected insights into the deep evolutionary history of viruses. However, only a minor subset of newly discovered viruses has been subject to full phenotypic characterization which can provide critical and fundamental insights into their biology and virus-host interactions, ultimately transforming our understanding of the evolutionary forces that shape the virosphere and disease emergence. Realistically, as important as these discoveries are to the advancement of science, very few of the viruses will ever cause human disease or influence the global economy.

This mass sequencing approach, which has been called viral metagenomics, can also be applied in a more targeted way to identify viruses in clinical cases of diseases of unknown etiology or to survey for potentially novel zoonotic viruses that may represent a significant risk of transmission to humans. Indeed, NGS is being used increasingly in conjunction with real-time PCR as a front-line tool in medical and veterinary settings for rapid detection and identification of exotic or unknown emerging viruses. For example, in 2009, an outbreak of acute hemorrhagic fever occurred in Mangala, Democratic Republic of Congo (DRC), involving three human cases, two of which were fatal. As no positive diagnosis was obtained using real-time PCR for known viral hemorrhagic fevers in Africa, NGS was conducted on acute phase serum collected from the surviving patient, revealing the near-complete sequence of a novel rhabdovirus, Bas-Congo virus (BASV; species *Bas Congo tibrovirus*) [260]. Although the disease was attributed by the investigators to the novel virus, no isolate was obtained and there was no evidence of neutralising antibodies in 43 serum samples from undiagnosed hemorrhagic fever cases or 50 random serum donors from the DRC. Subsequently, NGS of blood collected from healthy individuals from Nigeria identified two related viruses, Ekpoma virus 1 (EKV-1; species *Ekpoma 1 tibrovirus*) and Ekpoma virus 2 (EKV-2; species *Ekpoma 2 tibrovirus*), and a serological survey indicated that antibodies to these or similar viruses occur commonly in healthy humans in Nigeria [261]. However, once again, neither virus was isolated. Interestingly, several other tibroviruses had previously been isolated from healthy cattle or biting midges (*Culicoides* spp.) in Australia and Florida [79,83,262,263]. None have been associated with either disease in livestock or the infection of humans [83,264]. These and other studies raise important issues regarding the significance of NGS data, even when providing complete or near-complete viral genome sequences, when investigating disease etiology. Most viruses can cause asymptomatic infections and many newly discovered viruses may be benign in their natural host. Therefore, in the absence of a virus isolate which can be used for experimental studies, establishing a causal association with disease based only on detection of the viral genome should be approached cautiously.

### 4.2. The Taxonomic Challenge of Viral Biodiversity

The availability of a rapidly expanding number of novel viral genomes identified by metagenomic studies has also presented challenges for virus taxonomy—a system for classification of viruses that is administered by the International Committee on Taxonomy of Viruses (ICTV). Should viruses detected only by their nucleotide sequence be classified and assigned to species and other higher taxa (genus, family order, etc.) alongside viruses for which we have a viable isolate? The ICTV (then the International Committee on Nomenclature of Viruses) was established in 1966 at the ninth International Congress for Microbiology in Moscow, publishing its first report in 1971 [265]. It operates under the auspices of the International Union of Microbiological Societies (IUMS), with the authority to develop, refine, and maintain a universal virus taxonomy. Historically, the description and classification of a new virus by the ICTV required significant information, such as host range, serology, replication cycles, and structure, aspects that could be determined from the study of isolates. On the other hand, sequences alone provide a trove of information, including evolutionary relationships (e.g., phylogeny), genome organization (e.g., the number of genes and their order), presence or absence of distinctive motifs (e.g., protein cleavage sites, terminal sequences, internal ribosome entry sites), as well as genome composition (e.g., codon usage, GC content), which of course could be used to inform classification into species. These concerns framed the contents of a workshop of experts and members of the ICTV, resulting in a seminal consensus statement in which viruses identified only from metagenomic data are considered to be bona fide viruses and thus candidates for taxonomic assignment [266]. The expanding diversity of the known virosphere also presented challenges. A recent analysis of metagenomes of 3042 geographically and ecologically diverse samples led to the discovery of 125,842 new partial dsDNA viral genomes encoding more than 2.79 million proteins, 75% of which had no sequence similarity to proteins from known virus isolates [267]. Other metagenomic studies have revealed similar diversity in ssDNA and RNA viruses, particularly in marine ecosystems [268]. This has led ICTV to consider a far broader framework for taxonomic assignment of viruses, recently approving the establishment of a taxonomic hierarchy that includes 15 ranks (realm, subrealm, kingdom, subkingdom, phylum, subphylum, class, subclass, order, suborder, family, subfamily, genus, subgenus, and species), thus expanding the range even beyond those currently available for other organisms [269]. The sheer volume of new virus genomes identified by metagenomic studies has also led to the development of new bioinformatic tools, that are increasingly being applied for automated virus classification of viruses, based almost exclusively on nucleotide sequence data [270,271,272,273,274,275,276,277].

## 5. Recent Advances in Virus Characterization

While the classic tools of virus discovery and characterization (e.g., EM, serology, and tissue culture) are still widely used, NGS allowed for the rapid identification of an enormous repertoire of viruses, thus exponentially expanding the boundaries of the known virosphere. The resultant genomic sequences allowed for their accurate taxonomic assignments, analysis of their phylogenetic and evolutionary relationships with other viruses, and evaluation of the potential risks they may present to humans and wild or domestic animal populations. Below are a few representative examples of how NGS transformed the known relationships of arboviruses within their respective families.

### 5.1. Rhabdoviruses

Rhabdoviruses contain negative-sense (-) single-stranded RNA (ssRNA) genomes. They are amongst the most numerous and diverse of RNA viruses, naturally infecting mammals, birds, fish, reptiles, and amphibians, as well as insects, arachnids, crustaceans, nematodes, and a wide range of plants [278,279]. Most (but not all) rhabdoviruses that infect vertebrates are transmitted by hematophagous arthropods. The *Rhabdoviridae* currently comprises 20 genera containing 143 species and one unassigned species [280]. Of these, viruses assigned to seven of the genera (*Vesiculovirus*, *Ephemerovirus*, *Tibrovirus*, *Hapavirus*, *Ledantevirus*, *Curiovirus*, and *Sripuvirus*) are considered to be arboviruses. Viruses assigned to the newly characterized genus *Almendravirus* appear to be insect-specific [281]. Viruses assigned to the genus *Tupavirus* have been isolated only from vertebrates but may possibly have arthropod vectors. Complete coding sequences are now available for more than 100 other rhabdoviruses, many of which have been isolated from or detected in arthropods, but they have not yet been formally classified. While the classic tools of viral discovery (e.g., EM, serology, and tissue culture) are still widely used, NGS has played a central role in recent genome sequencing efforts, revealing diversity, not only in the ecology of rhabdoviruses but also in the structural diversity of genome architecture [252,282,283,284,285,286,287]. In addition to the five canonical rhabdovirus structural protein genes (N, P, M, G, and L), it is now recognized that rhabdoviruses commonly contain multiple long open reading frames encoding putative accessory proteins, mostly of unknown function. NGS has also facilitated studies of the evolution of rhabdovirus genome organization [252] and revealed the importance of arthropods in the evolutionary history of rhabdoviruses [257].

### 5.2. Bunyaviruses

According to the current International Committee on Taxonomy of Viruses (ICTV), classification of the order *Bunyavirales* encompasses 10 families of segmented (-) ssRNA viruses—*Arenaviridae*, *Hantaviridae*, *Nairoviridae*, *Peribunyaviridae* and *Cruliviridae*, *Mypoviridae*, *Fimoviridae*, *Wupedeviridae*, *Phasmaviridae*, and *Phenuiviridae*—a classification based on structural, genetic, and antigenic characteristics [288]. *Bunyavirales* is a diverse order with a large number of viruses associated with human, veterinary, and plant disease, as well as being vectored by arthropods (mosquitoes, ticks, sandflies, and thrips) and infecting a wide range of other invertebrates. The recent discovery of Gouléako (GOLV) [254] and Cumuto (CUMV) [289] viruses (the latter with NGS) which are evolutionarily related to but distinct from viruses in the genus *Phlebovirus, family* *Phenuiviridae*, suggesting they are to be assigned to the new genus *Goukovirus*. In the past five years, NGS has revolutionized understanding of the vast diversity of this order, by allowing genetic identification of previously uncharacterized and unassigned bunyaviruses, which in turn provided a complimentary approach to the gold standard of classification (structural, genetic, and antigenic characteristics) for a more refined taxonomic classification. Combined, these approaches will be instrumental for enhancing our understanding of their ecologic and geographic distribution, as well as public health impact.

### 5.3. Flaviviruses

The family *Flaviviridae* comprises positive-sense (+) ssRNA viruses. The family contains several serious human pathogens, including dengue, yellow fever, Zika, Japanese encephalitis, West Nile, and tick-borne encephalitis viruses (all arboviruses in the genus *Flavivirus*) and the hepatitis C virus (a member of the genus *Hepacivirus*). Members of the genus *Flavivirus*, like the alphaviruses (see Section 5.4), are a diverse group of arthropod-borne viruses that are found on every continent except Antarctica. Several flaviviruses have been discovered and characterized recently, mainly through chance or increased surveillance efforts, and facilitated through NGS. Newly characterized viruses include the Mercadeo virus (MECDV), from pools of *Culex species* mosquitoes in Panama [290], Sabethes flavivirus (SbFV), from *Sabethes belisarioi* in Brazil [291], the La Tina virus (LTNV), from *Aedes scapularis* in Peru [292], the Long Pine Key virus (LPKV), from *Anopheles crucians* in the Florida everglades, and the Kampung Karu virus (KPKV), from *Anopheles tesselatus* in Borneo [292], Aedes flavivirus (AeFV), from *Aedes albopictus* laboratory colony that originated in Thailand [293], Xishuangbanna flavivirus (XFV), from *Aedes albopictus* in China [294], and the Cuacua virus (CuCuV), from *Mansonia ssp*. in Mozambique [295].

One unexpected finding was the discovery of segmented viruses that grouped in the family *Flaviviridae* and were more closely related to flaviviruses than members of other established genera. The Jingmen tick virus (JMTV) is unique in the family *Flaviviridae* with a four segment positive-sense genome [296]. Traditional Sanger sequencing generated the NS3 and NS5 gene sequences but could not connect these within a single genome segment. Phylogenies based on either NS3-like or NS5-like sequences, which are encoded on two of the four segments, showed JMTV falling basal on the flavivirus tree and clearly distinct from the other viruses in this genus. The remaining two segments did not appear to have a flavivirus origin. JMTV has been isolated from cattle, monkeys, and ticks (*Rhipicephalus and Haemaphysalis* spp.) [297]. This prototype virus has lent its name to the newly identified segmented flaviviruses, and other viruses have recently populated the Jingmenvirus clade. Similarly, the Guaico Culex virus (GCXV) consists of five segments, and initial studies suggested GCXV is insect-specific, based on its isolation from *Culex ssp*. mosquitoes [298]. Additional potentially insect-specific four-segmented viruses include Shuangao insect virus 7 (SAIV7), the Wuhan flea virus (WHFV), Wuhan aphid virus 1 (WHAV1), Wuhan aphid virus 2 (WHAV2), and the Wuhan cricket virus (WHCV) [299]. Interestingly, sequences that are similar to *Toxocara canis*, a larva roundworm, and tentatively named T. canis larva agent virus (TCLAV), are distantly related to JMTV and appear to be the first evidence of a flavivirus-line organism in a member of the phylum Nematoda [296]. The discovery and characterization of a segmented genome flavivirus has significant implications, as it reveals that RNA virus segmentation is an evolutionary process that has occurred in previously unanticipated circumstances.

### 5.4. Alphaviruses

The alphaviruses are a diverse group of (+) ssRNA arthropod-borne viruses that are found on every continent except Antarctica [300]. There were no known mosquito-only alphaviruses until 2012, when analysis by NGS of a mosquito-pool from the Negev desert in Israel showed the presence of an alphavirus, the Eilat virus (EILV) [301]. Further analysis showed that, although EILV could infect mosquito cells, it was unable to replicate in vertebrate cells [302]. EILV is considered a vaccine candidate, as it can produce immunity without replication in the vertebrate host, illustrating the importance of such discoveries, which can lead to novel platforms for the prevention and/or treatment of disease [303].

### 5.5. Reoviruses

Reoviruses are a diverse family of double-stranded RNA (dsRNA) viruses that infect a wide range of hosts and have a wide range of characteristics. Of the 30 genera, there are only three for which novel viruses have been described using NGS, and the majority of these are in the genus *Orbivirus*. Interestingly, because of the structure of the genomes of reoviruses and the conserved sequences at the 5’ and 3’ ends of the segments [304], it is relatively simple to sequence segments of reoviruses using more traditional techniques. This may be why there are so few novel reoviruses determined by NGS. Novel viruses identified using NGS have been assigned to four other genera (*Seadornavirus*, *Orthoreovirus,* *Dinovernavirus,* and *Cypovirus*), all of which are shown in Table 1.

### 5.6. Negeviruses

Newly recognized viruses containing a (+) ssRNA genome have been proposed to form a new genus *Negevirus*, related to genera of mite-infecting plant viruses (*Blunervirus*, *Cilevirus*, and *Higrevirus*) in the new family *Kitaviridae* [251,253]. Originally, six viruses with restricted host range in insects (ISVs), designated as Negev (NEGV), Ngewotan (NWTV), Piura (PIUV), Loreto (LORV), Dezidougou (DEZV), and Santana (SANV), were identified in and isolated from mosquitoes and phlebotomine sandflies, collected in Brazil, the Ivory Coast, Israel, Indonesia, Peru, and the USA, [251]. Their widespread geographic distribution was documented by several other groups, who reported the isolation and characterization of related viruses in the Philippines (Tanay virus; TANAV) [315], Trinidad and Tobago (Wallerfield virus; WALV) [289], Côte d’Ivoire (Goutanap virus; GANV) [253], Portugal (Ochlerotatus caspius negevirus; OCNV and Culex univittatus negevirus; CUNV) [316], Brasil (Brajeira and Wallerfield viruses), Colombia, and Nepal. Collectively, the close relationship of negeviruses with plant viruses of the genera *Cilevirus*, *Higrevirus,* and *Blunervirus,* coupled with their heterogenous genome organization and architecture, provides support for the possibility that negeviruses are plant-like viruses that could eventually anchor a new virus family [317].

### 5.7. Mesoniviruses

Members of the family *Mesoniviridae*, a newly discovered family of (+) ssRNA viruses assigned to the order *Nidovirales* [318,319], appear to have an extensive geographic distribution but a restricted host range within members of the family Culicidae (flies) [251,318,319,320,321,322,323,324,325]. The *Mesoniviridae* comprise of a single genus *Alphamesonivirus* with nine recognized species: *Alphamesonivirus 1*, including Nam Dinh (NDiV) [318], Cavally (CavV) [319], NDiV A12.2520 [324], NDiV Ngewotan, and NDiV Houston [326] viruses; *Alphamesonivirus 2*, including the single isolate of the Karang Sari (KSaV) virus, and the four Bontag Baru (BBaV) isolates sampled in the early 1980s in Indonesia, but only recently characterized [326]; *Alphamesonivirus 3*, including the Dak Nong virus (DKNV), the three isolates of Kamphaeng Phet (KPhV), sampled in Indonesia and Thailand in the mid-1980s [322,326]; *Alphamesonivirus 4*, including the Casuarina virus (CASV), isolated in Australia from *Coquillettidia xanthogaster* mosquitoes [323]; *Alphamesonivirus 5*, the African Hana virus (HanaV) [320]; *Alphamesonivirus 6* and 7, including Ofaie (OFAV) and Kadiweu (KADV) viruses, isolated in the Pantanal of Brasil from *Mansonia* sp. mosquitoes, respectively [327]; and *Alphamesonivirus-8* and *9*, including the African Nse (NseV), and Meno (MenoV) viruses [320]; and the distinct yet unassigned species, the Yichang virus [325], isolated from *Culex sp.* mosquitoes in China.

## 6. Conclusion

Across several generations, virus hunters have left a profound legacy, both to science and to the broader global community. In the field, their work has often been conducted in difficult, demanding, and sometimes dangerous circumstances. In the laboratory, they have developed and applied tools and methodologies that led to improved diagnosis, prevention, and treatment of viral disease, and still lie at the center of virology today. Others working at more fundamental scientific levels have used their virus isolates as scientific models, opening the door to a revolution in molecular and cellular biology. Yet, their work continues. Viral disease emergence remains one of the most serious threats to humanity, with potentially devastating social and economic consequences. Recent examples, such as SARS, MERS, henipa-, Ebola, and Zika viruses, illustrate the threat that unidentified or poorly characterized viruses can, and almost certainly will, continue to present. Equally devastating emerging diseases of livestock, fisheries, and crops threaten food production and livelihoods. As history has repeatedly shown us, technological revolutions have been often accompanied by periods of progressive disinvestment in training for the eclipsed technologies of the past, and in our case, classical virology. The recent emergence of the Zika virus in the Americas has reinforced the notion that traditional methods of virus discovery and new technologies offer a complimentary toolkit, especially in resource-poor settings that can be seamlessly integrated in the service of public health. It is our hope and expectation that new generations of virus hunters will appreciate the legacy of their predecessors and complement the power of NGS, metagenomics, and bioinformatics, not only to advance viral evolutionary biology, but to understand and limit the potential impacts of emerging viral disease.

## Figures and Tables

**Table 1:** Novel reoviruses identified recently using various next generation sequencing (NGS) platforms.

Genus	Virus	Year^1^	NGS Platform	Reference
*Orbivirus*	Big Cypress virus*	2017	Illumina	[305]
	Ninarumi virus*	2017	Illumina	[305]
	High Island virus*	2017	Illumina	[305]
	bluetongue virus type 3	2017	Illumina	[306]
	Parry’s Lagoon virus*	2016	Illumina	[73]
	Irituia virus*	2013	Roche GS	[307]
	Mobuck virus*	2014	Ion Torrent	[308]
	Sathuvachari virus*	2013	Roche GS	[120]
	Tribec virus	2012	Roche GS	[309]
	Kemerovo virus	2012	Roche GS	[309]
*Orthoreovirus*	Mahlapitsi virus*	2016	Illumina	[310]
	largemouth bass reovirus*	2016	Illumina	[311]
*Seadornavirus*	Kadipiro virus	2016	Illumina	[312]
*Dinovernavirus*	Fako virus*	2015	Illumina	[313]
*Cypovirus*	Anopheles cypovirus*	2016	Illumina	[314]

^1^ Refers to year of the publication and not of the isolation of the virus. *Not yet formally classified.

## Supplementary Materials

Supplementary materials can be found at https://www.mdpi.com/1999-4915/11/5/471/s1.
