# Supplementary material for: Exploiting the Legacy of the Arbovirus Hunters
Source: Viruses. 2019 May 23;11(5):471. doi: 10.3390/v11050471 (PMC6563318; doi:10.3390/v11050471)
Supplement: Supplementary file 1 [file viruses-11-00471-s001.pdf]

Table 1. Arboviruses and emerging viral pathogens isolated for the first time in Australia, 1950-2018.

Virus	Isolate	Classification			First isolation			Reference
		Family	Genus	Species	Year	Location	Source	
Edge Hill virus	C281	*Flaviviridae*	*Flavivirus*	*Edge Hill virus*	1961	Cairns, QLD	mosquitoes (*Aedes vigilax + Culex annulirostris*)	Doherty et al AJEB 1963
Gadgets Gulley virus	CSIRO122	*Flaviviridae*	*Flavivirus*	*Gadgets Gully virus*	1975	Macquarie Island, TAS	hard ticks (*Ixodes uraie*)	St. George et al AJTMH 1985
Kokobera virus	MRM32	*Flaviviridae*	*Flavivirus*	*Kokobera virus*	1960	Kowanyama, QLD	mosquitoes (*Culex annulirostris*)	Doherty et al AJEB 1963
Stratford virus	C338	*Flaviviridae*	*Flavivirus*	*Kokobera virus*	1961	Cairns, QLD	mosquitoes (*Aedes vigilax*)	Doherty et al AJEB 1963
New Mapoon virus	CY1014	*Flaviviridae*	*Flavivirus*	*Kokobera virus**	1998	Cape York, QLD	mosquito sp.	Nisbet et al JGV 2005
Murray Valley encephalitis virus	152	*Flaviviridae*	*Flavivirus*	*Murray Valley encephalitis virus*	1951	Mooroopna, VIC	human	French MJA 1952
Alfuy virus	MRM3929	*Flaviviridae*	*Flavivirus*	*Murray Valley encephalitis virus*	1966	Kowanyama, QLD	swamp pheasant (*Centropus phasianinus*)	Whitehead et al TRSTMH 1968
Saumarez Reef virus	CSIRO4	*Flaviviridae*	*Flavivirus*	*Saumarez Reef virus*	1974	Saumarez Reef, QLD	soft ticks (*Ornithodoros capensis*)	St. George et al AJEBMS 1977
Kunjin virus	MRM16	*Flaviviridae*	*Flavivirus*	*West Nile virus*	1960	Kowanyama, QLD	mosquitoes (*Culex annulirostris*)	Doherty et al AJEB 1963
Finch Creek virus	EB6	*Nairoviridae*	*Nairovirus**	unassigned	2001	Macquarie Island, TAS	hard ticks (*Ixodes uraie*)	Major et al PLoS One 2009
Vinegar Hill virus	CSIRO1499	*Nairoviridae*	*Nairovirus**	unassigned	1981	Gatton, QLD	soft ticks (*Argas robertsi*)	Gauci et al Viruses 2017
Upolu virus	C5581	*Orthomyxoviridae*	*Thogotovirus**	unassigned	1966	Upolu Cay, QLD	soft ticks (*Ornithodoros capensis*)	Doherty et al AJS 1969
Cedar virus	CG1a	*Paramyxoviridae*	*Henipavirus*	*Cedar henipavirus*	2009	Cedar Grove, QLD	fruit bats (*Pteropus* sp.)	Marsh et al PLoS Path 2012
Hendra virus		*Paramyxoviridae*	*Henipavirus*	*Hendra henipavirus*	1994	Brisbane, QLD	horses (*Equus ferus caballus*)	Murray et al Science 1995
Menangle virus		*Paramyxoviridae*	*Rubulavirus*	*Menangle rubulavirus*	1997	NSW	pigs (*Sus scrofa domesticus*)	Philbey et al EID 1998
J virus		*Paramyxoviridae**	unassigned	unassigned	1972		mouse (*Mus musculus*)	Jun et al AJEBMS 1977
Mossman virus		*Paramyxoviridae**	unassigned	unassigned	1970	Mossman, QLD	rodent (*Rattus leucopus*)	Campbell et al Search 1977
Belmont virus	R8659	*Peribunyaviridae*	*Orthobunyavirus*		1968	Rockhampton, QLD	mosquitoes (*Culex annulirostris*)	Doherty et al AVJ 1972
Douglas virus	CSIRO150	*Peribunyaviridae*	*Orthobunyavirus*	*Sathuperi virus*	1978	Douglas Station, QLD	bovine (*Bos indicus*)	St George et al AJEBMS 1979
Facey's Paddock virus	Ch16129	*Peribunyaviridae*	*Orthobunyavirus*	*Oropouche virus*	1974	Charleville, QLD	mosquitoes (*Culex annulirostris*)	Doherty et al AJEBMS 1979
Gan Gan virus	NB6057	*Peribunyaviridae*	*Orthobunyavirus**	unassigned	1970	Nelson Bay, NSW	mosquitoes (*Aedes vigilax*)	Gard et al AJTMH 1973
Koongol virus	MRM31	*Peribunyaviridae*	*Orthobunyavirus*	*Koongol orthobunyavirus*	1960	Kowanyama, QLD	mosquitoes (*Culex annulirostris*)	Doherty et al AJEB 1963
Kowanyama virus	MRM1178	*Peribunyaviridae*	*Orthobunyavirus**	unassigned	1964	Kowanyama, QLD	mosquitoes (*Anopheles annulipes*)	Doherty et al TRSTMH 1968a
Leanyer virus	NT16701	*Peribunyaviridae*	*Orthobunyavirus*		1974	Leanyer, NT	mosquitoes (*Anopheles meraukensis*)	Doherty et al AJEBMS 1977
Little Sussex virus	Ch19546	*Peribunyaviridae*	*Orthobunyavirus*		1976	Charleville, QLD	mosquitoes (*Culex annulirostris*)	Doherty et al AJEBMS 1979
Maputta virus	MRM186	*Peribunyaviridae*	*Orthobunyavirus**	unassigned	1960	Kowanyama, QLD	mosquitoes (*Anopheles meraukensis*)	Doherty et al AJEB 1963
Parker's Farm virus	Ch19520	*Peribunyaviridae*	*Orthobunyavirus*		1976	Charleville, QLD	mosquitoes (*Culex annulirostris*)	Doherty et al AJEBMS 1979
Peaton virus	CSIRO133	*Peribunyaviridae*	*Orthobunyavirus*	*Shamonda virus*	1976	Peachester, QLD	biting midges (*Culicoides brevitatsis*)	St George et al AJEBMS 1979
Taggert virus	MI14850	*Peribunyaviridae*	*Orthobunyavirus*	*Sakhalin orthonairovirus*	1972	Macquarie Island, TAS	hard ticks (*Ixodes uraie*)	Doherty et al AJTMH 1975
Termeil virus	BP8090	*Peribunyaviridae*	*Orthobunyavirus**	unassigned	1972	Bawley Point, NSW	mosquitoes (*Aedes camptorhynchus*)	Marshall et al AJEBMS 1980
Tinaroo virus	CSIRO153	*Peribunyaviridae*	*Orthobunyavirus*	*Akabane virus*	1978	Kiari, QLD	biting midges (*Culicoides brevitatsis*)	St George et al AJEBMS 1979
Trubanaman virus	MRM3630	*Peribunyaviridae*	*Orthobunyavirus**	unassigned	1966	Kowanyama, QLD	mosquitoes (*Anopheles annulipes*)	Doherty et al TRSTMH 1968a
Wongal virus	MRM168	*Peribunyaviridae*	*Orthobunyavirus*	*Koongol orthobunyavirus*	1960	Kowanyama, QLD	mosquitoes (*Culex annulirostris*)	Doherty et al AJEB 1963
Yacaaba virus	NB6028	*Peribunyaviridae*	*Orthobunyavirus**	unassigned	1970	Nelson Bay, NSW	mosquitoes (*Aedes vigilax*)	Gard et al AJTMH 1973
Precarious Point virus	MI19334	*Phenuiviridae*	*Phlebovirus*	*Uukuniemi phlebovirus*	1975	Macquarie Island, TAS	hard ticks (*Ixodes uraie*)	St. George et al AJTMH 1985
Catch-me-cave virus	I2/19	*Phenuiviridae*	*Phlebovirus**	unassigned	2001	Macquarie Island, TAS	hard ticks (*Ixodes uraie*)	Major et al PLoS One 2009
bluetongue virus type 20	CSIRO19	*Reoviridae*	*Orbivirus*	*Bluetongue virus*	1975	Beatrice Hill, NT	biting midges (*Culicoides* spp.)	St George et al AVJ 1978
bluetongue virus type 21	CSIRO154	*Reoviridae*	*Orbivirus*	*Bluetongue virus*	1979	Victoria River, NT	bovine (*Bos indicus*)	St George et al AVJ 1980
Corriparta virus	MRM1	*Reoviridae*	*Orbivirus*	*Corriparta virus*	1960	Kowanyama, QLD	mosquitoes (*Culex annulirostris*)	Doherty et al AJEB 1963
Parry's Lagoon virus	K75749	*Reoviridae*	*Orbivirus*	*Corriparta virus**	2010	Wyndham, WA	mosquitoes (*Culex annulirostris*)	Harrison et al Viruses 2016
Eubenangee virus	In1074	*Reoviridae*	*Orbivirus*	*Eubenangee virus*	1963	Innisfail, QLD	mosquitoes (mixed pool)	Doherty et al TRSTMH 1968b
Tilligerry virus	NB7080	*Reoviridae*	*Orbivirus*	*Eubenangee virus*	1971	Nelson Bay, NSW	mosquitoes (*Anopheles annulipes*)	Gard et al AJTMH 1973
Nugget virus	MI14847	*Reoviridae*	*Orbivirus*	*Kemerovo virus*	1972	Macquarie Island, TAS	hard ticks (*Ixodes uraie*)	Doherty et al AJTMH 1975
Bunyip Creek virus	CSIRO58	*Reoviridae*	*Orbivirus*	*Palyam virus*	1976	Grafton, NSW	bovine (*Bos taurus*)	Cybinski & St. George 1982
CSIRO Village virus	CSIRO11	*Reoviridae*	*Orbivirus*	*Palyam virus*	1974	Beatrice Hill, NT	biting midges (*Culicoides* spp.)	Cybinski & St. George 1982
D'Aguilar virus	B8112	*Reoviridae*	*Orbivirus*	*Palyam virus*	1968	Bunya, QLD	biting midges (*Culicoides brevitatsis*)	Doherty et al AVJ 1972
Marrakai virus	CSIRO82	*Reoviridae*	*Orbivirus*	*Palyam virus*	1975	Beatrice Hill, NT	biting midges (Culicoides *schultzei* + C. *peregrinus*)	Cybinski & St. George 1982
Mudjinbarry virus	NT14952	*Reoviridae*	*Orbivirus*	*Wallal virus*	1971	Alligator Rivers, NT	biting midges (*Culicoides marksi*)	Doherty et al AJBS 1978
Wallal virus	Ch11963	*Reoviridae*	*Orbivirus*	*Wallal virus*	1970	Charleville, QLD	biting midges (*Culicoides dycei + C. marksi*)	Doherty et al AVJ 1972
Mitchell River virus	MRM10434	*Reoviridae*	*Orbivirus*	*Warrego virus*	1969	Kowanyama, QLD	biting midges (*Culicoides* spp.)	Doherty et al AVJ 1972
Warrego virus	Ch9935	*Reoviridae*	*Orbivirus*	*Warrego virus*	1969	Charleville, QLD	biting midges (*Culicoides* spp.)	Doherty et al AVJ 1972
Paroo River virus	GG668	*Reoviridae*	*Orbivirus*	*Wongorr virus*	1973	Wanaaring, NSW	mosquitoes (*Culex annulirostris*)	Marshall et al AJEBMS 1982
Picola virus	PK886	*Reoviridae*	*Orbivirus*	*Wongorr virus*	1974	Picola, VIC	mosquitoes (*Culex annulirostris*)	Marshall et al AJEBMS 1982
Wongorr virus	MRM13443	*Reoviridae*	*Orbivirus*	*Wongorr virus*	1970	Kowanyama, QLD	mosquitoes (*Aedes lineatopennis*)	Doherty et al AVJ 1972
Lake Clarendon virus	CSIRO704	*Reoviridae*	*Orbivirus**	unassigned	1980	Gatton, QLD	soft ticks (*Argas robertsi*)	St George et al AJBS 1984
Middle Point virus	DPP4440	*Reoviridae*	*Orbivirus**	unassigned	1998	Beatrice Hill, NT	bovine (*Bos indicus*)	Cowled et al JGV 2007

Sandy Bay virus	F3/4	*Reoviridae*	*Orbivirus**	unassigned	2001	Macquarie Island, TAS	hard ticks (*Ixodes uraie*)	Major et al PLoS One 2009
Stretch Lagoon virus	K49460	*Reoviridae*	*Orbivirus**	unassigned	2002	Wolfe Creek, WA	mosquitoes (*Culex annulirostris*)	
Nelson Bay virus	NB847	*Reoviridae*	*Orthoreovirus*	*Nelson Bay orthoreovirus*	1968	Nelson Bay, NSW	fruit bat (*Pteropus poliocephalus*)	Gard & Marshall ARCH VIROL 1973
Adelaide River virus	DPP61	*Rhabdoviridae*	*Ephemerovirus*	*Adelaide River ephemerovirus*	1981	Tortilla Flats, NT	bovine (*Bos indicus*)	Gard et al AVJ 1984
Berrimah virus	DPP63	*Rhabdoviridae*	*Ephemerovirus*	*Berrimah ephemerovirus*	1981	Darwin, NT	bovine (*Bos indicus*)	Gard et al AVJ 1983
Hayes Yard virus	DPP4816	*Rhabdoviridae*	*Ephemerovirus**	unassigned	2000	Beatrice Hill, NT	bovine (*Bos indicus*)	Blasdell et al in prep
Kimberley virus	OR250	*Rhabdoviridae*	*Ephemerovirus*	*Kimberley ephemerovirus*	1973	Kunnanurra, WA	mosquitoes (*Culex annulirostris*)	Liehne et al AJEBMS 1981
Koolpinyah virus	DPP819	*Rhabdoviridae*	*Ephemerovirus*	*Koolpinyah ephemerovirus*	1985	Darwin, NT	bovine (*Bos indicus*)	Gard et al Intervirology 1992
Holmes Jungle virus	DPP1163	*Rhabdoviridae*	*Hapavirus**	unassigned	1987	Darwin, NT	mosquitoes (*Culex annulirostris*)	Gubala et al Evol Bioinformat 2017
Ngaingan virus	MRM14556	*Rhabdoviridae*	*Hapavirus*	*Ngaingan hapavirus*	1970	Kowanyama, QLD	biting midges (*Culicoides brevitatsis*)	Doherty et al AVJ 1972
Ord River virus	OR1023	*Rhabdoviridae*	*Hapavirus*	*Ord River hapavirus*	1976	Kunnanurra, WA	mosquitoes (*Culex annulirostris*)	
Parry's Creek virus	OR189	*Rhabdoviridae*	*Hapavirus*	*Parry Creek hapavirus*	1973	Kunnanurra, WA	mosquitoes (*Culex annulirostris*)	Liehne et al AJEBMS 1981
Wongabel virus	CSIRO264	*Rhabdoviridae*	*Hapavirus*	*Wongabel hapavirus*	1979	Wongabel, QLD	biting midges (*Culicoides austropalpalis*)	Gubala et al Virology 2008
Australian bat lyssavirus		*Rhabdoviridae*	*Lyssavirus*	*Australian bat lyssavirus*	1996	Ballina, NSW	fruit bat (*Pteropus alecto*)	Fraser et al EID 1996
Almpiwar virus	MRM4059	*Rhabdoviridae*	*Sripuvirus*	*Almpiwar sripuvirus*	1966	Kowanyama, QLD	skink (*Cryptoblepharus virgatus*)	
Humpty Doo virus	CSIRO79	*Rhabdoviridae*	*Sripuvirus**	unassigned	1975	Beatrice Hill, NT	sandflies (*Lasiohelia* spp.)	Standfast et al AJBS 1984
Beatrice Hill virus	CSIRO25	*Rhabdoviridae*	*Tibrovirus*	*Beatrice Hill tibroirus*	1975	Beatrice Hill, NT	biting midges (*Culicoides peregrinus*)	Standfast et al AJBS 1984
Coastal Plains virus	DPP53	*Rhabdoviridae*	*Tibrovirus*	*Coastal Plains tibrovirus*	1981	Palmerston, NT	bovine (*Bos taurus*)	Cybinski & Gard AJBS 1986
Tibrogargan virus	CSIRO132	*Rhabdoviridae*	*Tibrovirus*	*Tibrogargan tibrovirus*	1976	Peachester, QLD	biting midges (*Culicoides brevitatsis*)	Cybinski et al Vet Microbiol 1980
Harrison Dam virus	CSIRO75	*Rhabdoviridae**	unassigned	unassigned	1975	Beatrice Hill, NT	mosquitoes (*Culex annulirostris*)	Standfast et al AJBS 1984
Kunnanurra virus	OR194	*Rhabdoviridae**	unassigned	unassigned	1973	Kunnanurra, WA	mosquitoes (*Aedeomyia catasticta*)	Liehne et al AJEBMS 1981
Oak Vale virus	K13965	*Rhabdoviridae**	unassigned	unassigned	1993	Kununurra, WA	mosquitoes (*Anopheles annulipes*)	Quan et al Virus Res 2011
Walkabout Creek virus	CSIRO1056	*Rhabdoviridae**	unassigned	unassigned	1981	Samford, QLD	biting midges (*Culicoides austropalpalis*)	McAllister et al Virol Rep 2014
Barmah Forest virus	BH2193	*Togaviridae*	*Alphavirus*	*Barmah Forest virus*	1974	Barmah Forest, VIC	mosquitoes (*Culex annulirostris*)	Marshall et al AJEBMS 1982
Murweh virus	Ch16313	*Togaviridae*	*Alphavirus*	*Barmah Forest virus*	1974	Charleville, QLD	mosquitoes (*Culex annulirostris*)	Doherty et al AJEBMS 1979
Ross River virus	T48	*Togaviridae*	*Alphavirus*	*Ross River virus*	1959	Townsville, QLD	mosquitoes (*Aedes vigilax*)	Doherty et al AJS 1963
Charleville virus	Ch9824	unassigned			1969	Charleville, QLD	sandfly (*Phelbotomus* sp.)	Doherty et al AVJ 1972
Samford virus = Aino virus								

*presumptive member
